# Diel variations in planktonic ciliate community structure in the northern South China Sea and tropical Western Pacific

**DOI:** 10.1038/s41598-023-30973-6

**Published:** 2023-03-08

**Authors:** Chaofeng Wang, Yi Dong, Michel Denis, Yuanyuan Wei, Haibo Li, Shan Zheng, Wuchang Zhang, Tian Xiao

**Affiliations:** 1 CAS Key Laboratory of Marine Ecology and Environmental Sciences, Institute of Oceanology, Chinese Academy of Sciences, Qingdao, 266071 China; 2 Laboratory for Marine Ecology and Environmental Science, Qingdao National Laboratory for Marine Science and Technology, Qingdao, 266237 China; 3 Center for Ocean Mega-Science, Chinese Academy of Sciences, Qingdao, 266071 China; 4 Aix Marseille Université, Université de Toulon, CNRS/INSU, IRD, Institut Méditerranéen d’Océanologie (MIO), 13288 Marseille Cedex 09, France; 5 College of Marine Ecology and Environment, Shanghai Ocean University, Shanghai, 201306 China; 6 Jiaozhou Bay Marine Ecosystem Research Station, Institute of Oceanology, Chinese Academy of Sciences, Qingdao, 266071 China

**Keywords:** Microbial ecology, Microbial ecology

## Abstract

Though diel variations are geographically widespread phenomena among phytoplankton and zooplankton, knowledge is limited regarding diel variations in planktonic ciliate (microzooplankton) community structure. In this study, we analyzed diel variations in community structure of planktonic ciliates in the northern South China Sea (nSCS) and tropical Western Pacific (tWP). Hydrological characteristics during day and night were slightly different over both the nSCS and tWP, while ciliate average abundance at night was clearly higher than in the day in the upper 200 m. In both the nSCS and tWP, abundance proportions of large size-fraction (> 30 μm) aloricate ciliates at night were higher than in the day. While for tintinnids, abundance proportion of large lorica oral diameter at night were lower than in the day. The relationship between environmental factors and ciliate abundance pointed out that depth and temperature were main factors influencing aloricate ciliate and tintinnid in both day and night. For some dominant tintinnid species, chlorophyll *a* was another important factor influencing their diel vertical distribution. Our results provide fundamental data for better understanding the mechanisms of planktonic ciliate community diel variation in the tropical Western Pacific Ocean.

## Introduction

Planktonic ciliates taxonomically belong to phylum Ciliophora, class Spirotrichea, subclass Oligotrichia and Choreotrichia^1^, and they morphologically consist of tintinnids and aloricate ciliates. Marine planktonic ciliates are important components of microzooplankton as primary consumers of pico- (0.2–2 μm) and nano- (2–20 μm) sized plankton, and important food items of metazoans and fish larvae^2–4^. Therefore, they play an important role in material circulation and energy flow from the microbial food web into the classical food chain^5–7^. Owing to their rapid growth rates and sensitivity to environmental changes, ciliates, especially tintinnids, have been considered as effective bioindicators in different water masses because of distinctive species composition^8–10^.

Diel variations, which are common phenomenon in marine plankton, include variations in abundance, behavior, physiology, feeding and cell-division^11–15^. The diel behavior of phytoplankton was found to be affected by light-dependence of cell growth and continuous losses to grazing in the tropical and subtropical seas^12,16–18^, which eventually led to community diel variations. For example, in the northern South China Sea (nSCS) at night, the abundance and cell size of picophytoplankton (*Prochlorococcus*, *Synechococcus*, and picoeukaryotes) were respectively higher and smaller than during the day^18^. With respect to marine planktonic zooplankton, most studies dedicated to meso-/macro-zooplankton, which have higher abundance at night than in the day owing to their diel vertical migration (descending at dawn and ascending in late afternoon and evening)^19–26^.

In contrast, studies related to planktonic ciliate (microzooplankton) diel variations remain limited, even though several investigations on planktonic-ciliate diel variations were conducted in different habitats^27–35^. In oceanic waters, the mixotrophic ciliate *Mesodinium rubrum* was shown higher abundance at surface waters at daytime than at night in the Baltic Sea^30,32^. Some micro-sized heterotrophic ciliates at night were more abundant at surface water than in the day in the northwestern Mediterranean Sea^33^. Those above two phenomenon about diel variations owing to their different diel vertical migration behaviors. But in the shelf and slope waters of the Georges Bank (northwest Atlantic)^29^, and the Toyama Bay (Japan Sea)^31^, abundance of planktonic ciliates varied little during the day and night. In the eutrophic shallow waters of a Germany gravel pit lake characterised by stable water stratification, Rossberg and Wickham^34^ found that the abundances of several dominant ciliate species were significantly higher in the day than at night. Despite their important role in marine microbial food webs, our knowledge of ciliate assemblage diel variations in tropical oceanic waters are limited due to their inaccessibility for oceanographic surveys.

The South China Sea is the largest semi-enclosed basin in the western Pacific Ocean^36^, and the tropical Western Pacific (tWP) holds the largest warm pool area with sea-surface temperature > 28 °C throughout the year^37^. Many studies were conducted on ciliate communities in the northern slope of the South China Sea^9,38–41^ and the tWP^4,8,42–44^. However, none of these studies addressed ciliate community diel variations, nor provided any comparison between the nSCS (marginal oceanic sea) and tWP (tropical oceanic sea).

In the present study, we hypothesized that planktonic ciliate community structure might differ between day and night. By examining time-series data of ciliate community structure in the nSCS and tWP, we aimed to determine diel variations in: (1) ciliate abundance and biomass at each sampled depth; (2) overall abundance and abundance proportions of different size-fractions of aloricate ciliates; (3) tintinnid composition and the abundance proportions of different lorica oral diameter (LOD) size-classes. The output of this study is expected to be of great help in monitoring microzooplankton ecological influence in the marginal and tropical oceanic seas.

## Results

### Hydrology and ciliate vertical distribution diel variations

**Figure 1:**
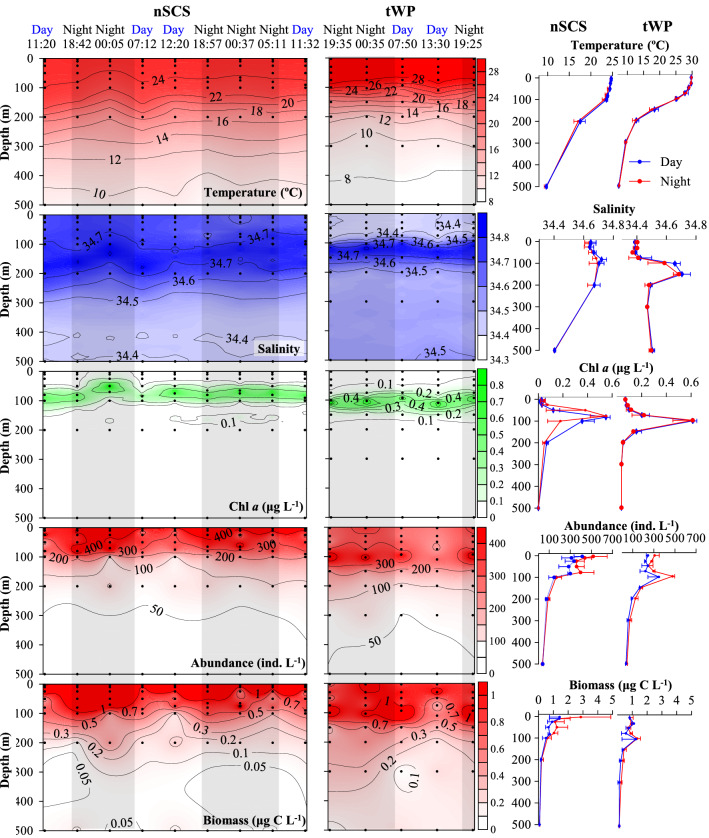
Temperature (T), salinity (S), Chlorophyll *a* (Chl *a*), total ciliate abundance and biomass profiles from the surface to 500 m in the northern South China Sea (nSCS) and tropical Western Pacific (tWP). Black dots: sampling depths; black shadows: night.

Hydrological characteristics throughout day and night were slightly different in the nSCS and tWP (Fig. 1). Temperature decreased with depth from surface (3 m) to 500 m. However from surface to 100 m depth at nSCS, its average values at each depth at daytime were slightly higher by 0.20 ± 0.16 °C than at night. In contrast, in the tWP, even temperature values at 30, 150 and 200 m were higher at daytime than at night, the average temperature values at each depth at daytime were slightly lower by 0.24 ± 0.26 °C than at night (Fig. 1). Salinity first increased from surface to approximately 150 m, then decreased to 500 m in both the nSCS and the tWP. Salinity average values at depths from surface to 100 m at daytime at both the nSCS and the tWP, were slightly higher by 0.01 ± 0.01 and 0.01 ± 0.03, respectively, than at night. At each depth, salinity in the nSCS was higher than in tWP (Fig. 1). Chlorophyll *a *in vivo fluorescence (Chl *a*) showed similar characteristics in both day and night, while average deep Chl *a* maximum (DCM) layers in the day of the nSCS (82.5 ± 6.5 m) and the tWP (101.5 ± 12.0 m) were deeper than at night (nSCS: 77.0 ± 4.5 m; tWP: 98.7 ± 5.1 m), respectively (Fig. 1).

High ciliate total abundance (≥ 200 ind. L^−1^) and biomass (≥ 0.5 μg C L^−1^) values were mainly observed in the upper 100 m of nSCS and the upper 150 m of tWP, and the values decreased down to 500 m depth (Fig. 1). Aloricate ciliates were dominant groups in both the nSCS and tWP (Supplementary Fig. S1). The vertical profiles of ciliate average abundance showed bimodal (in the surface and DCM layers) patterns throughout day and night in both the nSCS and tWP. But average biomass showed surface-peak in the nSCS and DCM-peak in the tWP, respectively (Fig. 1). PERMANOVA tests indicated no significant differences between day and night of total ciliate abundance data in both the nSCS (pseudo-*F* = 0.074, *P* = 0.944) and tWP (pseudo-*F* = 0.609, *P* = 0.494) (Supplementary Table S1). From surface to 200 m depth, average abundance and biomass of ciliates at night were higher than in the day in the nSCS. While in the tWP, only average abundance of ciliates at night were higher than in the day.

Highest average total abundance and biomass values of ciliates in the nSCS occurred in surface layers, whereas in the tWP, they occurred in the DCM layers (Fig. 1). At surface layers of the nSCS, average abundance (517.0 ± 132.6 ind. L^−1^) and biomass (2.8 ± 2.0 μg C L^−1^) at night were 1.3 and 2.0 folds higher than in the day (413.3 ± 77.6 ind. L^−1^ and 1.4 ± 0.7 μg C L^−1^), respectively. At DCM layers of the tWP, average abundance (476.7 ± 21.4 ind. L^−1^) and biomass (1.3 ± 0.2 μg C L^−1^) at night were 1.4 and 1.1 folds higher than in the day (347.0 ± 103.2 ind. L^−1^ and 1.2 ± 0.9 μg C L^−1^), respectively (Fig. 1). There were almost no differences between day and night in waters deeper than 200 m in the nSCS and tWP, respectively (Fig. 1; Supplementary Fig. S1).

### Water column average abundance and biomass of ciliates

**Figure 2:**
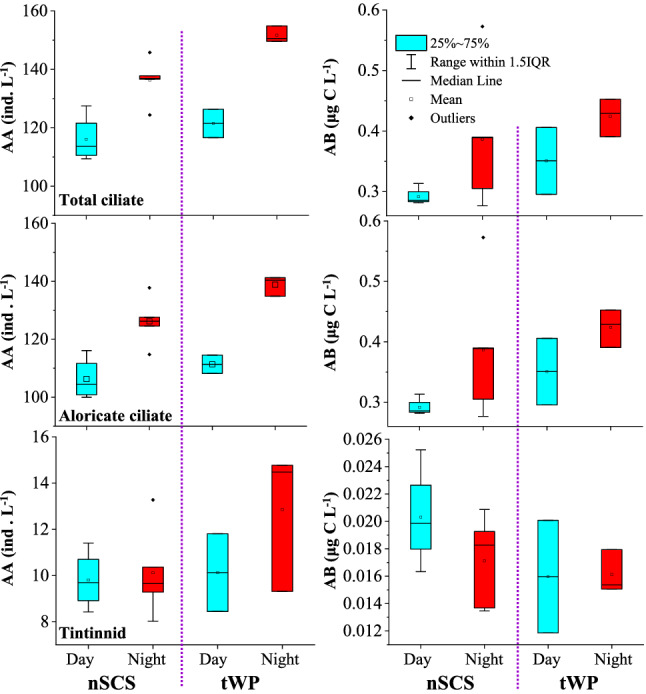
Diel variations of ciliate (total, aloricate ciliate and tintinnid) water column average abundance (AA) and average biomass (AB) in the nSCS and tWP.

Average abundance and biomass of ciliate showed different characteristics during day and night in both the nSCS and tWP (Fig. 2). In the nSCS, the average water-column abundance of total ciliates (136.3 ± 7.7 ind. L^−1^) (Independent *t*-test, *P* < 0.01) and aloricate ciliates (126.2 ± 8.2 ind. L^−1^) (Independent *t*-test, *P* < 0.01) at night were significant higher than that in the day (116.1 ± 8.1 ind. L^−1^, and 106.3 ± 7.3 ind. L^−1^), respectively. As for tintinnids, the average water-column abundance at night (10.1 ± 2.0 ind. L^−1^) were slight higher than that in the day (9.8 ± 1.2 ind. L^−1^) (Independent *t*-test, *P* ≥ 0.05), but the average water-column biomass of tintinnids was lower at night (0.017 ± 0.003 μg C L^−1^) than in the day (0.020 ± 0.004 μg C L^−1^).

In the tWP, the average water-column abundance and biomass of total ciliates, aloricate ciliates and tintinnids at night were higher than in the day (Fig. 2). As to variations between two seas, the average water-column abundance and biomass of total ciliates and aloricate ciliates at night and day were higher in the tWP than that in the nSCS (Fig. 2), but not significant (Independent *t*-test, *P* ≥ 0.05). Although average water-column abundance of tintinnids in both the night and day in the tWP were higher than that in the nSCS, their average water-column biomass were lower in the tWP than in the nSCS (Fig. 2).

### Aloricate ciliate size-fractions

**Figure 3:**
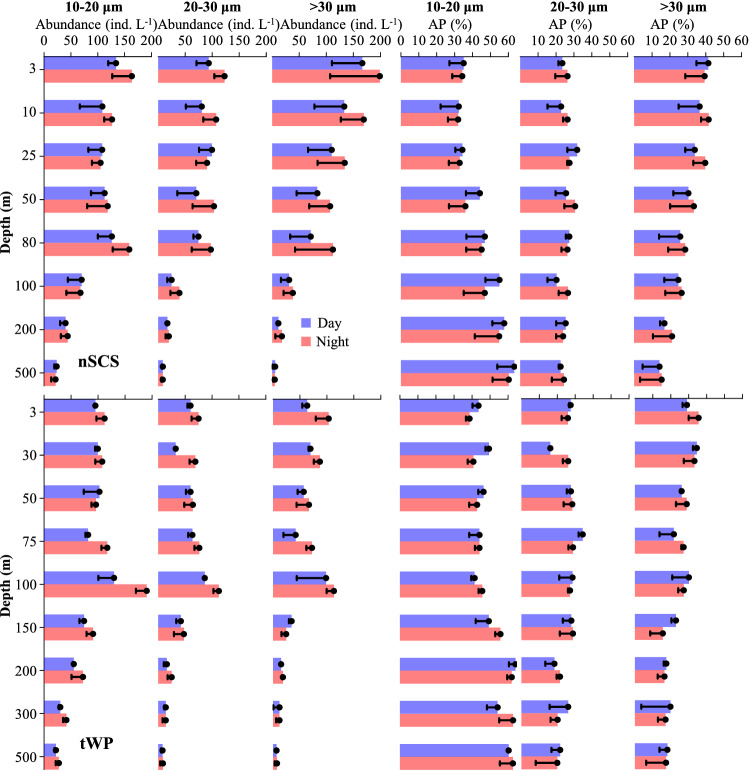
Diel variations of average abundance and abundance proportion (AP) of each aloricate ciliate size-fraction at each layers in the nSCS and tWP.

In the nSCS and tWP, day and night average abundance and abundance proportion of each aloricate ciliate size-fraction, were different (Fig. 3). Generally, in the upper 150 m of both the nSCS and tWP, average abundance of small (10–20 μm), medium (20–30 μm) and large (> 30 μm) size-fractions of aloricate ciliate were higher at night than that in the day. In contrast, in the nSCS upper 150 m and tWP upper 75 m, abundance proportions of the small size-fraction were lower at night than that in the day. As to day and night variations in approximately the upper 80 m of nSCS and tWP, the average abundance and abundance proportion of the large size-fraction of aloricate ciliates were higher in the nSCS than that in the tWP. However, the opposite was observed at 100 m (Fig. 3). There was almost no difference between day and night abundance and abundance proportion in waters deeper than 200 m in both the nSCS and tWP (Fig. 3).

### Tintinnid abundance, composition, and diversity index

In total, 69 tintinnid species from 27 genera were identified through the study (Supplementary Table S2). Among them, 57 species from 23 genera and 51 species from 25 genera were observed at the nSCS and tWP, respectively. Species richness at night over the nSCS (49 species) and tWP (45 species) were slightly higher than in the day (nSCS: 44 species, tWP: 44 species), respectively (Supplementary Table S2). Tintinnid abundance ranged from 0 to 87 ind. L^−1^ and 0–73 ind. L^−1^ in the nSCS and tWP, respectively. Both high abundance (≥ 10 ind. L^−1^) and species richness (≥ 5) occurred in the upper 200 m. In the nSCS, Margalef (*d*_*Ma*_) and Shannon (*H′*) indices were higher at night than in the day. However, in the tWP, these diversity indices hardly varied from day to night (Supplementary Fig. 2). As for tintinnid biogeography type, cosmopolitan and warm water genera were the dominant groups at both sites. Regarding diel variations in both the nSCS and tWP, more cosmopolitan and warm water species were found at night than that in the day (Supplementary Table S3).

**Figure 4:**
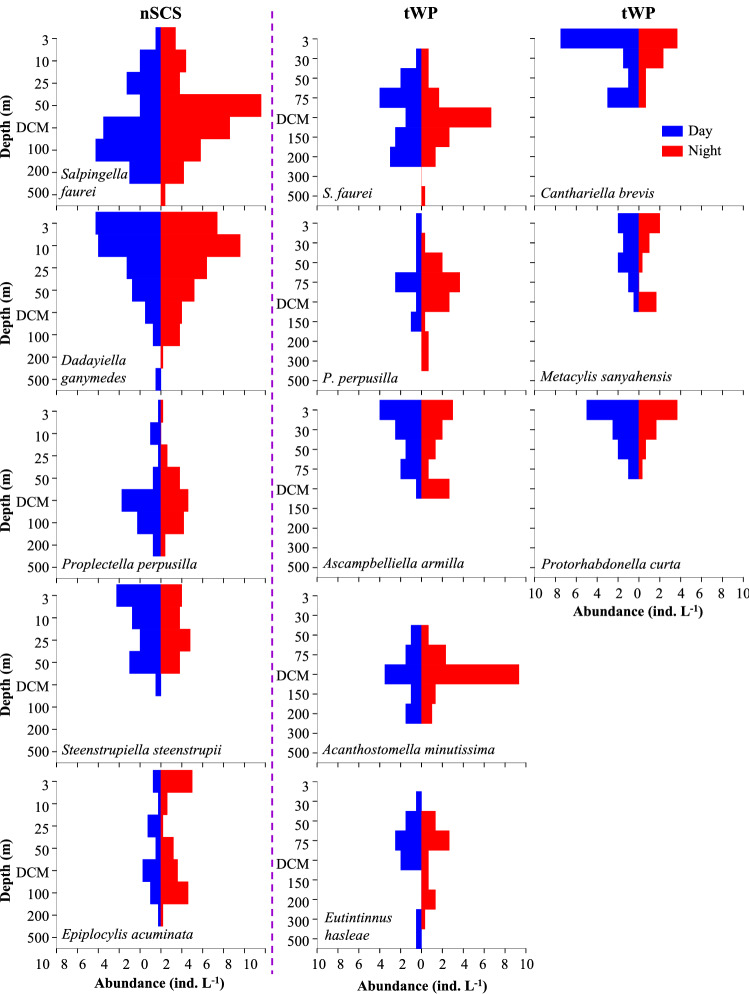
Diel variations of average abundance of tintinnid dominant species at each layer in the nSCS and tWP.

Five and eight dominant species (*Y* ≥ 0.02) occurred in the nSCS and tWP, respectively. Among them, only *Salpingella faurei* and *Proplectella perpusilla* appeared in both sites (Supplementary Table S2). As for dominant species in the nSCS, *S. faurei* and *Epiplocylis acuminata* prefer 50 m and DCM layers and their high abundance occurred at night more frequently. In contrast, in the surface layer, *Dadayiella ganymedes* and *Steenstrupiella steenstrupii* were present in higher abundance in the day than at night. The *P. perpusilla* prefer 25 m and 50 m layers at night. But at DCM and 100 m layers, its high abundance occurred in the day more frequently (Fig. 4; Supplementary Figs. S3 and S4). In the tWP, *S. faurei*, *P. perpusilla*, *Ascampbelliella armilla, Acanthostomella minutissima* and *Metacylis sanyahensis* prefer DCM layers and their high abundance occurred at night more frequently. While for *Canthariella brevis* and *Protorhabdonella curta*, their abundance were higher in the day than at night in surface layers. With regard to *Eutintinnus hasleae*, its abundance was higher at night than in the day in waters ranged from 50 to 200 m (except DCM) (Fig. 4; Supplementary Figs. S3 and S4).

### Body size composition and abundance proportion of tintinnid species

**Figure 5:**
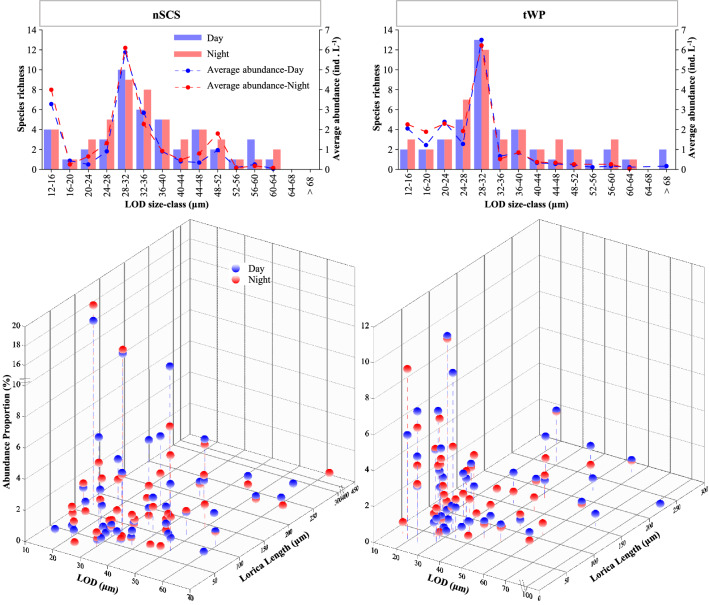
Day-night variations of tintinnid species richness, lorica oral diameter (LOD), lorica length, average abundance and abundance proportion in the nSCS and tWP.

Average abundance in tintinnid LOD (lorica oral diameter) size-classes had some differences throughout the day and night in both the nSCS and tWP (Fig. 5). Highest species richness and average abundance were in the 28–32 μm LOD size-class during the day and night in both the nSCS and tWP. For both day and night, the second highest species richness in the nSCS and tWP were 32–36 μm and 24–28 μm LOD size-class, respectively, while the second highest average abundance were 12–16 μm and 20–24 μm LOD size-class in the nSCS and tWP, respectively. Generally, average abundance of most tintinnid LOD size-classes were higher at night than in the day. However, these night and day values were similar in the tWP (Fig. 5).

In the nSCS, abundance proportion of *S. faurei* (highest, 16.8%) and *D. ganymedes* (second highest, 15.7%) were lower in the day than at night (18.4% and 16.1%, respectively). Abundance proportion of *S. steenstrupii* (third highest, 9.3%) was higher in the day than at night (5.5%). In the tWP, *S. faurei* (9.2%), *C. brevis* (8.8%) and *P. curta* (7.1%) had the three highest abundance proportion in the day. At night, however, species with the three highest abundance proportion changed to *A. minutissima* (9.5%), *S. faurei* (9.0%) and *P. perpusilla* (6.2%) (Fig. 5). Additionally, tintinnid species with lorica length greater than 150 μm had higher abundance proportion in the day than at night in both the nSCS and tWP (Fig. 5).

### Relationship between ciliate abundance and environmental factors

**Figure 6:**
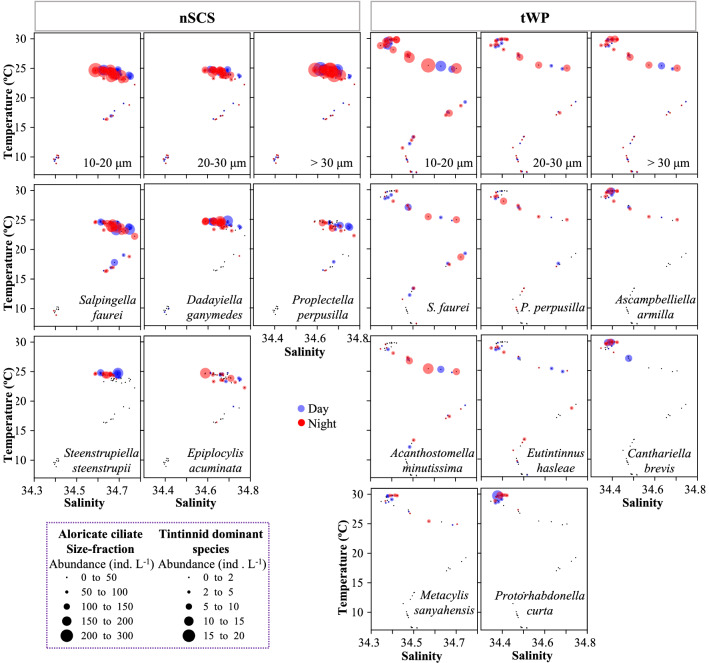
Temperature-salinity-plankton diagrams for day-night variations of size-fractions (10–20 μm, 20–30 μm and > 30 μm) of aloricate ciliate and tintinnid dominant species in the nSCS and tWP.

Temperature-salinity-plankton diagrams showed that aloricate ciliate size-fractions (small, medium, and large) and tintinnid dominant species behaved within different temperature and salinity ranges that varied from day and night in the nSCS and tWP (Fig. 6). Regarding differences between the two sites, the average temperature of each aloricate ciliate size-fraction with abundance > 100 ind. L^−1^ in the nSCS (23.1–24.8 °C, average 24.3 ± 0.5 °C) was lower than that in the tWP (24.8–29.8 °C, average 27.8 ± 1.9 °C) (Supplementary Fig. S5). As for tintinnids, all dominant species (except *D. ganymedes*) in the nSCS had temperature ranges wider at night than in the day, and their higher abundance was associated with salinity higher in the day (except *E. acuminata*) than at night (Fig. 6). In the tWP, all dominant species (except *S. faurei*) corresponded to wider salinity ranges at night than in the day (Fig. 6; Supplementary Fig. S6).

Relationships between ciliate abundances and environmental factors (depth, temperature, salinity, and Chl *a*) during day and night, differed in both the nSCS and tWP (Table 1; Supplementary Table S4). In the nSCS and tWP, Aloricate ciliates and total ciliates had strong significant negative and positive correlations with depth and temperature, respectively, whether in the day or at night. As for dominant tintinnids in the nSCS, *S. faurei* had significant positive correlation with Chl *a* at night, but no correlation with Chl *a* at daytime. *P. perpusilla* had significant positive correlation with Chl *a* in the day, but no correlation with Chl *a* at night (Supplementary Table S4). In the tWP, *S. faurei*, *P. perpusilla*, *M. sanyahensis* and total tintinnids were not correlated with Chl *a* in the day, but they exhibited significant correlations at night (Supplementary Table S4).

**Table 1:** Partial Mantel tests comparison between ciliate community (aloricate ciliate, tintinnid, and total ciliate) and environmental factors (depth, temperature, salinity, and Chl *a*).

Seas	Day/Night	Environmental factors	Aloricate ciliate		Tintinnid		Total ciliate	
			*P*	*R*	*P*	*R*	*P*	*R*
nSCS	Day	Depth	**0.001**	**0.803**	0.322	0.034	**0.001**	**0.867**
	Day	Temperature	**0.001**	**0.810**	0.430	− 0.002	**0.001**	**0.877**
	Day	Salinity	**0.001**	**0.560**	0.384	0.019	**0.001**	**0.682**
	Day	Chl *a*	0.969	− 0.106	**0.040**	**0.186**	0.591	− 0.037
	Night	Depth	**0.001**	**0.855**	**0.045**	**0.155**	**0.001**	**0.843**
	Night	Temperature	**0.001**	**0.865**	0.055	0.139	**0.001**	**0.843**
	Night	Salinity	**0.001**	**0.634**	0.201	0.069	**0.001**	**0.670**
	Night	Chl *a*	0.762	− 0.069	**0.047**	**0.151**	0.498	− 0.020
tWP	Day	Depth	**0.001**	**0.822**	0.140	0.151	**0.001**	**0.859**
	Day	Temperature	**0.001**	**0.814**	**0.003**	**0.360**	**0.001**	**0.802**
	Day	Salinity	0.624	− 0.048	0.077	0.177	0.759	− 0.081
	Day	Chl *a*	0.124	0.174	0.317	0.040	0.219	0.109
	Night	Depth	**0.001**	**0.847**	0.477	− 0.014	**0.001**	**0.832**
	Night	Temperature	**0.001**	**0.838**	0.054	0.129	**0.001**	**0.739**
	Night	Salinity	0.575	− 0.032	0.059	0.190	0.477	− 0.015
	Night	Chl *a*	0.070	0.176	**0.001**	**0.524**	**0.013**	**0.328**

Numbers in bold indicate statistically significant results.

The partial Mantel test revealed that aloricate ciliate and total ciliate had similar significant correlations with their each environmental factors between day and night in both the nSCS and tWP, respectively. But different for tintinnid. In the nSCS, except Chl *a*, tintinnid had significant correlation with depth at night (*P* < 0.05). In the tWP, tintinnid had significant correlation with temperature in the day (*P* < 0.05), but changed into had strong significant correlation with Chl *a* at night (*P* < 0.01) (Table 1).

## Discussion

### Diel vertical distribution variations of ciliate community

In oceans, there were multiple factors that could influence diel variations of ciliate (diel-vertical-migration, food items concentration and quality, predator avoidance, light intensity, body metabolic rates, etc.) in various seas^27,33,34,45–47^. Previous studies found that most planktonic ciliates do not show perceivable vertical migration^29,31^, thus we speculate that diel-vertical-migration might not be an determining factor for ciliate diel variations. In the oligotrophic seas, the phytoplankton assemblage was dominated by *Prochlorococcus*, *Synechococcus* and picoeukaryotes, and they showed different diel variations^12,48^. As important food items of ciliate, heterotrophic bacteria also displayed clear daily oscillations in the oligotrophic Ionian Sea (Mediterranean)^48^. Thus we speculate that diel variation of food items was possibly the main reason in determining ciliate diel variation in the oligotrophic tropical seas.

The ciliate abundance was high in surface and DCM layers in both day and night of both the nSCS and tWP. These results were similar to previous ones established in the western Pacific Ocean^40,42–45,49^ and eastern Indian Ocean^50^. However, the studies that previously investigated the ciliate vertical distribution, did not assess potential differences between day and night in vertical direction. Therefore, our study provides more accurate data on ciliate diel variation in the nSCS and tWP. Additionally, our results in the upper 200 m provide evidence that ciliate abundance were higher at night than in the day in both the nSCS and tWP (Figs. 1 and 2). Zooplankton distribution in waters mainly depends on phytoplankton presence^51,52^. Thus, it is possible that the availability of more food items (flagellates, picoeukaryotes, *Prochlorococcus*, *Synechococcus* and heterotrophic bacteria) at night than in the day explains the higher ciliate abundance at night^18,32,48^.

### Diel variations in aloricate ciliate size-fractions

Abundance proportions of different aloricate ciliate size-fractions have rarely been reported in the nSCS and tWP. In the tropical Pacific Ocean, average abundance proportions of small size-fraction (10–20 μm) of aloricate ciliates to total ciliates ranged from 38 to 50% (from surface to 200 m depth), and it belonged to the dominant group at each depth in most stations^43,44,49^. Our results for the small size-fraction of aloricate ciliates in the tWP are consistent with those of previous studies in both day and night. In the upper 100 m of both nSCS and tWP sites, the large size-fraction (> 30 μm) of aloricate ciliates had more pronounced diel variations than those of the small size-fraction (Fig. 3). We speculated that the large size-fraction of aloricate ciliates were migrating along distances longer than those crossed by the small size-fraction. This phenomenon may be similar to that observed in meso-/macro-zooplankton in the nSCS^25^, equatorial Pacific Ocean^53^, subtropical and subarctic North Pacific Ocean^54^, and northwest Mediterranean^55^.

### Potential reason for tintinnid diel variations

The LOD of a tintinnid is closely related to the size of its preferred food item (approximately 25% of the LOD)^56^. Our results showed that tintinnid abundance was higher but biomass was lower at night than in the day in both the nSCS and tWP (Fig. 2). We also found that abundance and abundance proportion of the 12–16 μm LOD size-class of tintinnids was higher at night than in the day. These results suggest that both LOD size-classes of tintinnids and the size of their preferred food items were smaller at night than in the day. The night-dominant smaller cell sizes of food items (picoeukaryotes, *Prochlorococcus*, *Synechococcus*) at night than in the day^18^ may be coupled with the observed tintinnid diel variations.

For photosynthetic organisms, cell division generally occurs at night and/or in the late afternoon^17,57^, which eventually leads to higher abundance at night than in the day^18^. As for heterotrophic microzooplankton tintinnids, photosynthetic organisms, e.g., nanoplankton (nanoflagellates), are important food items influencing their abundance and composition in the oligotrophic seas^58,59^. Our study showed that tintinnid abundance at night was higher than in the day for two possible reasons: (1) oceanic tintinnid species have stronger cell division in midnight than in the day in tropical Pacific waters^60^; and (2) predation on picoplankton, nanoplankton and heterotrophic bacteria occurred primarily at night^61–63^. Further studies on growth rates and cell division of tintinnid species are needed to better characterizing their diel vertical migration in the Pacific Ocean.

### Differences of ciliate community between the nSCS and tWP oceanic waters

Abundance peaks of planktonic ciliates occurred in surface and DCM layers in both the nSCS and tWP, but highest abundances occurred in surface layer of the nSCS, and DCM layer of the tWP (Fig. 1). Our results are consistent with Wang et al.^40^, which discovered this phenomenon and proposed a hypothesis to verify it. The nSCS is located at the convergence area of the shelf and slope waters where exchanges often occur with nutrient loaded waters originating from the Pearl River through surface current^64–71^. For example, the nutrient values in the Pearl River (~ 100 μM) were about 100 folds higher than in the nSCS slope (~ 1.2 μM)^66,70,71^. Nutrients are material basis for the growth of microphytoplankton and heterotrophic bacteria^68^. High nutrient concentrations always accompanied with high abundance of microphytoplankton and heterotrophic bacteria in surface waters in the oligotrophic tropical seas, which further affected and determined microzooplankton abundance and composition^72–76^. In contrast, the tWP is located at a tropical Pacific warm pool surrounded year-round by oligotrophic oceanic water. This may be the main reason for the surface layer ciliate abundance in the nSCS clearly higher than in the tWP.

Aloricate ciliates were dominant groups at each sampled depth of both sites (Supplementary Fig. S1), which was similar to previous observations in adjacent seas^4,9,40,42,44,49^. As for tintinnid assemblages, we identified more species in the nSCS (57 species) than in the tWP (51 species) (Supplementary Table S2), which was not consistent with previous investigations^40,43,77^, who found more species in adjacent seas. Low sampling frequency is often accompanied by low species richness^78,79^. The total samples in the tWP (45 samples) and nSCS (72 samples) were much lower than in previous studies (≥ 100 samples)^40,43,77^. Thus we speculate that low sampling frequency in our results could be the main reason for the disagreement. High tintinnid abundance and species richness mainly appeared at around DCM depths in both the nSCS and tWP. A high Chl *a* environment may be an important factor for influencing tintinnid distribution in oceanic waters^80,81^.

## Methods

### Study area and sample collection

**Figure 7:**
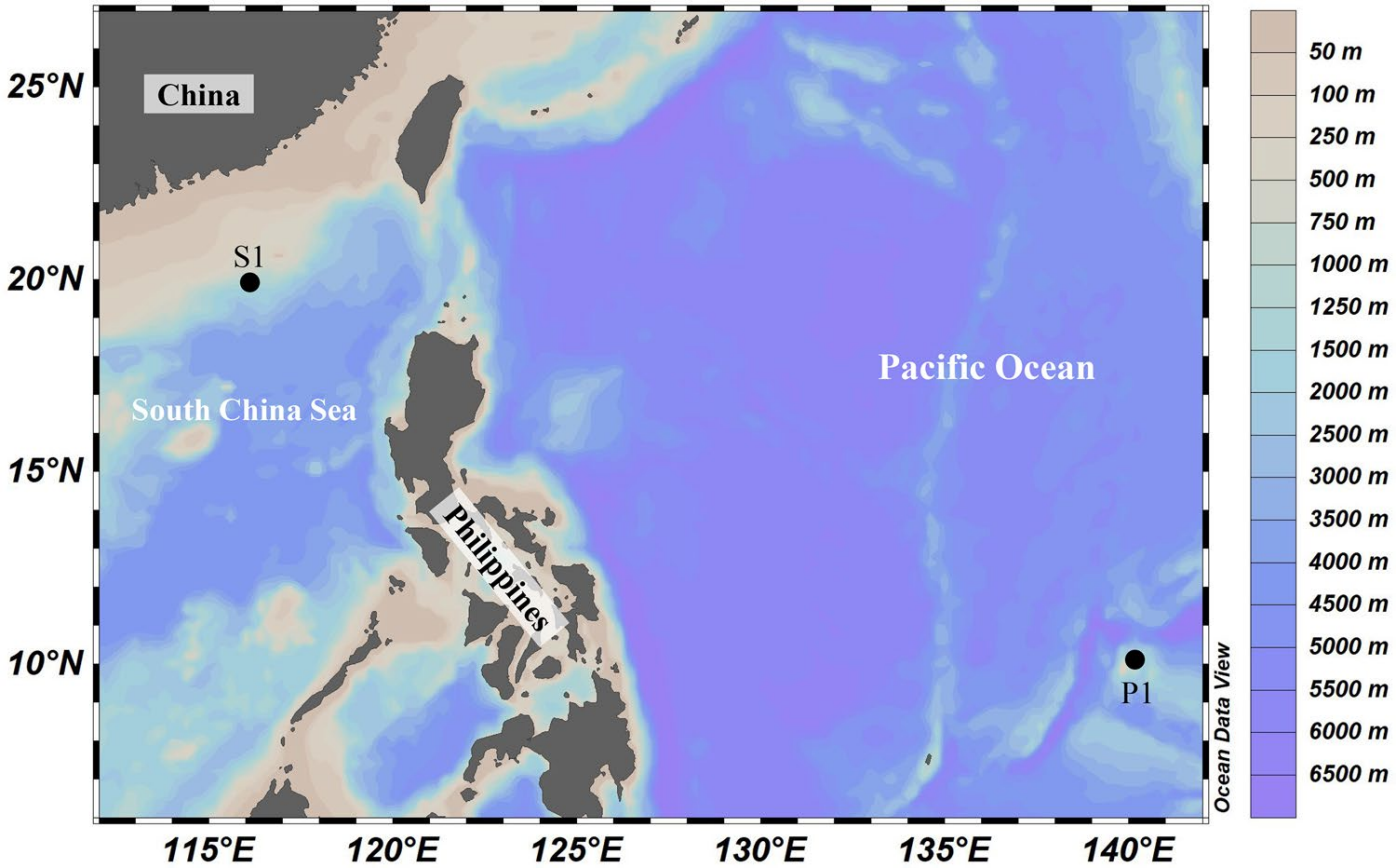
Survey stations in the northern South China Sea (nSCS) and tropical West Pacific (tWP).

The variation of ciliate vertical distribution was addressed by conducting two time-series sampling in the upper 500 m at two distinct sites, Station (St.) S1 in nSCS and St. P1 in tWP, during two different cruises (Fig. 7). St. S1 was visited from 29 to 31 March 2017 aboard R.V. “Nanfeng”, and St. P1 from 2 to 3 June 2019 aboard R.V. “Kexue”. During 48 h (St. S1) or 24 h (St. P1) sampling periods, seawater samples were collected by using a CTD (Sea-Bird Electronics, Bellevue, WA, USA)—rosette carrying 12 Niskin bottles of 12 L each (Supplementary Table S5). In the nSCS, the sampling depths were 3, 10, 25, 50, DCM (deep Chl a maximum layer), 100, 200 and 500 m; in the tWP, the sampling depths were surface (3), 30, 50, 75, DCM, 150, 200, 300 and 500 m. Casts were approximately launched every 6 h, the CTD determining vertical profiles of temperature, salinity and chlorophyll *a *in vivo fluorescence (Chl *a*). A total of 117 seawater samples were collected for planktonic ciliate community structure analysis. For each depth, 1 L seawater sample was fixed with acid Lugol’s (1% final concentration) and stored in darkness at 4 °C during the cruise.

### Sample analysis and species identification

In the laboratory, water samples were concentrated to approximately 200 mL by siphoning off the supernatant after the sample had settled for 60 h. This settling and siphoning process was repeated until a final concentrated volume of 50 mL was achieved, which was then settled in two Utermöhl counting chambers (25 mL per chamber)^82^ for at least 24 h. Planktonic ciliates were counted using an Olympus IX 73 inverted microscope (100 × or 400 ×) according to the process of Utermöhl^82^ and Lund et al.^83^.

For each species, size (length, width, according to shape) of the cell (aloricate ciliate) or lorica (tintinnid, especially length and oral diameter) were determined for at least 10 individuals if possible. Aloricate ciliates were categorized into small (10–20 μm), medium (20–30 μm) and large (> 30 μm) size-fractions for maximum body length of each individual following Wang et al.^43^. Tintinnid taxa were identified according to the size and shape of loricae following previous references^1,9,40,44,84–86^. Tintinnid species richness in each station was highlighted by the number of tintinnid species that appeared in that station. Because mechanical and chemical disturbance during collection and fixation can detach the tintinnid protoplasm from the loricae^87,88^, we included empty tintinnid loricae in cell counts.

### Data processing

Ciliate volumes were estimated using appropriate geometric shapes (cone, ball, and cylinder). Tintinnid carbon biomass was estimated using the equation^89^:

$$ C\, = \,V_{i} \, \times \,0.0{53}\, + \,{444}.{5,} $$

where *C* (μg C L^−1^) is the carbon biomass, *V*_*i*_ (μm^3^) is the lorica volume. We used a conversion factor of carbon biomass for aloricate ciliates of 0.19 pg/μm^3^^90^. The average abundance and biomass of the water column were calculated following Yu et al.^91^ and Wang et al.^92^. We used the Margalef index (*d*_*Ma*_)^93^ and Shannon index (*H′*)^94^ to test tintinnid diversity indices in the day and night variations. Biogeographically, the tintinnid genera are mainly classified into two groups in the oceanic waters based on Dolan and Pierce^95^: Cosmopolitan, species distributed widespread in the world ocean; Warm Water, species observed in both coastal systems and open waters throughout the world ocean, but absent from sub-polar and polar waters.

The dominance index (Y) of tintinnid species in one assemblage was calculated using formula^96^:

$$Y=\frac{{N}_{i}}{(N\times {f}_{i})},$$

where *N*_*i*_ is the number of individuals of species *i* in all samples, *f*_i_ is the occurrence frequency of species *i* in all samples and *N* is the total number of species. Species with *Y* ≥ 0.02 represented the dominant species in an assemblage.

Distributional data of sampling stations, ciliates and environmental parameters (Depth, temperature, salinity, and Chl *a*) were visualized by ODV (Ocean Data View, Version 5.0), Surfer (Version 13.0), OriginPro 2021 (Version 9.6), and Grapher (Version 12.0). Correlation analysis between environmental and biological variables (nonparametric-test, Independent *t*-test, Spearman’s rank analysis) were performed using SPSS (Version 16). The significance for grouping in the environment and ciliate community (aloricate ciliate and tintinnid) was tested by PERMANOVA analysis in PERMANOVA C of PRIMER 6^97,98^. The partial Mantel tests were performed between ciliate community and environmental factors in R4.1.1.

## Supplementary Information

Supplementary Information 1.

Supplementary Information 2.

## Data Availability

Datasets for this research are included in this paper and its supplementary information files (Supplementary Data).

## References

[1] Lynn DH (2008). Ciliated Protozoa: Characterization, Classification, and Guide to the Literature.

[2] Stoecker DK, Michaels AE, Davis LH (1987). Grazing by the jellyfish, *Aurelia aurita*, on microzooplankton. J. Plankton Res..

[3] Dolan JR, Vidussi F, Claustre H (1999). Planktonic ciliates in the Mediterranean Sea: Longitudinal trends. Deep-Sea Res..

[4] Gómez F (2007). Trends on the distribution of ciliates in the open Pacific Ocean. Acta Oecol..

[5] Azam F, Fenchel T, Field JG, Gray JS, Meyer-Reil LA, Thingstad F (1983). The ecological role of water column microbes in the sea. Mar. Ecol. Prog. Ser..

[6] Pierce RW, Turner JT (1992). Ecology of planktonic ciliates in marine food webs. Rev. Aquat. Sci..

[7] Calbet A, Saiz E (2005). The ciliate-copepod link in marine ecosystems. Aquat. Microb. Ecol..

[8] Kim YO, Shin K, Jang PG, Choi HW, Noh JH, Yang EJ (2012). Tintinnid species as biological indicators for monitoring intrusion of the warm oceanic waters into Korean coastal waters. Ocean Sci. J..

[9] Wang CF, Xu MQ, Xuan J, Li HB, Zheng S, Zhao Y (2021). Impact of the warm eddy on planktonic ciliate, with an emphasis on tintinnids as bioindicator species. Ecol. Indic..

[10] Wang CF, Wang XY, Xu ZQ, Hao Q, Zhao Y, Zhang WC (2022). Planktonic tintinnid community structure variations in different water masses of the Arctic Basin. Front. Mar. Sci..

[11] Haney JF (1988). Diel patterns of zooplankton behavior. Bull. Mar. Sci..

[12] Vaulot D, Marie D (1999). Diel variability of photosynthetic picoplankton in the equatorial Pacific. J. Geophys. Res-Oceans.

[13] Hays GC, Webb PI, Frears SL (1998). Diet changes in the carbon and nitrogen content of the copepod *Metridia lucens*. J. Plankton Res..

[14] Hays GC, Harris RP, Head RN (2001). Diel changes in the near-surface biomass of zooplankton and the carbon content of vertical migrants. Deep-Sea Res..

[15] Anna A, Enric S, Albert C (2020). Towards an understanding of diel feeding phythms in marine protists: Consequences of light manipulation. Microb. Ecol..

[16] Vaulot D, Marie D, Olson RJ, Chisholm SW (1995). Growth of *Prochlorococcus*, a photosynthetic prokaryote, in the equatorial Pacific Ocean. Science.

[17] Binder BJ, DuRand MD (2002). Diel cycles in surface waters of the equatorial Pacific. Deep-Sea Res..

[18] Li CL, Chiang KP, Laws EA, Liu X, Chen JX, Huang YB (2022). Quasi-antiphase diel patterns of abundance and cell size/biomass of picophytoplankton in the oligotrophic ocean. Geophys. Res. Lett..

[19] Ohman MD (1990). The demographic benefits of diel vertical migration by zooplankton. Ecol. Monogr..

[20] Ringelberg J (1999). The photo behavior of Daphnia spp. as a model to explain diel vertical migration in zooplankton. Biol. Rev..

[21] Tarling GA, Jarvis T, Emsley SM, Matthews JBL (2002). Midnight sinking behaviour in *Calanus finmarchicus*: A response to satiation or krill predation?. Mar. Ecol. Prog..

[22] Cohen JH, Forward RB (2005). Diel vertical migration of the marine copepod *Calanopia americana*. I. Twilight DVM and its relationship to the diel light cycle. Mar. Biol..

[23] Cohen JH, Forward RB (2005). Diel vertical migration of the marine copepod *Calanopia americana*. II. Proximate role of exogenous light cues and endogenous rhythms. Mar. Biol..

[24] Ringelberg J (2010). Diel Vertical Migration of Zooplankton in Lakes and Oceans.

[25] Liu HJ, Zhu ML, Guo SJ, Zhao XH, Sun XX (2020). Effects of an anticyclonic eddy on the distribution and community structure of zooplankton in the South China Sea northern slope. J. Mar. Syst..

[26] Tao ZC, Xian HC, Luan ZD, Nan F, Wang YQ, Song S (2022). The diel vertical distribution and carbon biomass of the zooplankton community in the Caroline Seamount area of the western tropical Pacific Ocean. Sci. Rep..

[27] Dale T (1987). Diel vertical distribution of planktonic ciliates in Lindåspollene, Western Norway. Mar. Microb. Food Webs.

[28] Jonsson PR (1989). Vertical distribution of planktonic ciliates–an experimental analysis of swimming behavior. Mar. Ecol. Prog. Ser..

[29] Stocker DK, Taniguchi A, Michaels AE (1989). Abundance of autotrophic, mixotrophic and heterotrophic ciliates in shelf and slope waters. Mar. Ecol. Prog. Ser..

[30] Passow U (1991). Vertical migration of *Gonyaulax catenata* and *Mesodinium rubrum*. Mar. Biol..

[31] Suzuki T, Taniguchi A (1997). Temporal change of clustered distribution of planktonic ciliates in Toyama Bay in summers of 1989 and 1990. J. Oceanogr..

[32] Olli K (1999). Diel vertical migration of phytoplankton and heterotrophic flagellates in the Gulf of Riga. J. Mar. Syst..

[33] Pérez MT, Dolan JR, Vidussi F, Fukai E (2000). Diel vertical distribution of planktonic ciliates within the surface layer of the NW Mediterrean (May 1995). Deep-Sea Res..

[34] Rossberg M, Wickham SA (2008). Ciliate vertical distribution and diel vertical migration in a eutrophic lake. Fund. Appl. Limnol..

[35] Gu BW, Huang H, Zhang YZ, Li R, Wang L, Wang Y (2022). High dynamics of ciliate community revealed via short-term, high-frequency sampling in a subtropical estuarine ecosystem. Front. Microbiol..

[36] Su JL (2004). Overview of the South China Sea circulation and its influence on the coastal physical oceanography near the Pearl River Estuary. Cont. Shelf Res..

[37] Cravatte S, Delcroix T, Zhang D, Mcphaden M, Leloup J (2009). Observed freshening and warming of the western pacific warm pool. Clim. Dyn..

[38] Feng MP, Zhang WC, Yu Y, Xiao T, Sun J (2013). Horizontal distribution of tintinnids in the western South China Sea during summer 2007. J. Trop. Oceanogr..

[39] Liu HX, Shen PP, Li CH, Chen ZZ, Qi ZH, Huang HH (2016). Composition and distribution of planktonic ciliates in the southern South China Sea during late summer: Comparison between surface and 75 m deep layer. J. Ocean Univ. China.

[40] Wang CF, Li HB, Zhao L, Zhao Y, Dong Y, Zhang WC (2019). Vertical distribution of planktonic ciliates in the oceanic and slope areas of the western Pacific Ocean. Deep-Sea Res..

[41] Sun P, Zhang SL, Wang Y, Huang BQ (2021). Biogeographic role of the Kuroshio Current Intrusion in the microzooplankton community in the boundary zone of the northern South China Sea. Microorganisms.

[42] Sohrin R, Imazawa M, Fukuda H, Suzuki Y (2010). Full-depth profiles of prokaryotes, heterotrophic nanoflagellates, and ciliates along a transect from the equatorial to the subarctic central Pacific Ocean. Deep-Sea Res..

[43] Wang CF, Li HB, Xu ZQ, Zheng S, Hao Q, Dong Y (2020). Difference of planktonic ciliate communities of the tropical West Pacific, the Bering Sea and the Arctic Ocean. Acta Oceanol. Sin..

[44] Wang CF, Li HB, Dong Y, Zhao L, Grégori G, Zhao Y (2021). Planktonic ciliate trait structure variation over Yap, Mariana and Caroline seamounts in the tropical western Pacific Ocean. J. Oceanol. Limnol..

[45] McLaren IA (1974). Demographic strategy of vertical migration by a marine copepod. Amer. Nat..

[46] Loose CJ, Von Elert E, Dawidowicz P (1993). Chemically-induced diel vertical migration in Daphnia: A new bioassay for kairomones exuded by fish. Arch. Hydrobiol..

[47] Bandara K, Varpe Ø, Wijewardene L, Tverberg V, Eiane K (2021). Two hundred years of zooplankton vertical migration research. Biol. Rev..

[48] Oubelkheir K, Sciandra A (2008). Diel variations in particle stocks in the oligotrophic waters of the Ionian Sea (Mediterranean). J. Mar. Syst..

[49] Yang EJ, Choi JK, Hyun JH (2004). Distribution and structure of heterotrophic protist communities in the northeast equatorial Pacific Ocean. Mar. Biol..

[50] Wang CF, Zhao YC, Du P, Ma X, Li SH, Li HB, Zhang WC, Xiao T (2022). Planktonic ciliate community structure and its distribution in the oxygen minimum zones in the Bay of Bengal (Eastern Indian Ocean). J. Sea Res..

[51] Daro MH (1988). Migratory and grazing behavior of copepods and vertical distribution of phytoplankton. Bull. Mar. Sci..

[52] Ursella L, Cardin V, Batistić M, Garić R, Gačić M (2018). Evidence of zooplankton vertical migration from continuous Southern Adriatic buoy current-meter records. Prog. Oceanogr..

[53] Roman MR, Dam HG, Le Borgne R, Zhang X (2002). Latitudinal comparisons of equatorial Pacific zooplankton. Deep-Sea Res..

[54] Steinberg DK, Cope JS, Wilson SE, Kobari T (2008). A comparison of mesopelagic mesozooplankton community structure in the subtropical and subarctic North Pacific Ocean. Deep-Sea Res..

[55] Isla A, Scharek R, Latasa M (2015). Zooplankton diel vertical migration and contribution to deep active carbon flux in the NW Mediterranean. J. Mar. Syst..

[56] Dolan JR (2010). Morphology and ecology in tintinnid ciliates of the marine plankton: Correlates of lorica dimensions. Acta Protozoologica.

[57] Jacquet S, Partensky F, Lennon JF, Vaulot D (2001). Diel patterns of growth and division in marine picoplankton in culture. J. Phycol..

[58] Pitta P, Giannakourou A, Christaki U (2001). Planktonic ciliates in the oligotrophic Mediterranean Sea: Longitudinal trends of standing stocks, distributions and analysis of food vacuole contents. Aquat. Microb. Ecol..

[59] Weisse T, Montagnes DJS (2022). Ecology of planktonic ciliates in a changing world: Concepts, methods, and challenges. J. Eukaryot. Microbiol..

[60] Heinbokel JF (1987). Diel periodicities and rates of reproduction in natural populations of tintinnines in the oligotrophic waters off Hawaii, September 1982. Mar. Microb. Food Webs.

[61] Tsai AY, Chiang KP, Chang J, Gong GC (2005). Seasonal diel variations of picoplankton and nanoplankton in a subtropical western Pacific coastal ecosystem. Limnol. Oceanogr..

[62] Ribalet F, Swalwell J, Clayton S, Jiménez V, Sudek S, Lin Y (2015). Light-driven synchrony of *Prochlorococcus* growth and mortality in the subtropical Pacific gyre. Proc. Natl. Acad. Sci. U. S. A..

[63] Connell PE, Ribalet F, Armbrust EV, White A, Caron DA (2020). Diel oscillations in the feeding activity of heterotrophic and mixotrophic nanoplankton in the North Pacific Subtropical Gyre. Aquat. Microb. Ecol..

[64] Cheung KC, Poon B, Lan CY, Wong MH (2003). Assessment of metal and nutrient concentrations in river water and sediment collected from the cities in the Pearl River Delta, South China. Chemosphere.

[65] Huang XP, Huang LM, Yue WZ (2003). The characteristics of nutrients and eutrophication in the Pearl River estuary. South China. Mar. Pollut. Bull..

[66] Liu SM, Guo X, Chen Q, Zhang J, Bi YF, Luo X (2010). Nutrient dynamics in the winter thermohaline frontal zone of the northern shelf region of the South China Sea. J. Geophys. Res..

[67] Shu YQ, Wang Q, Zu TT (2018). Progress on shelf and slope circulation in the northern South China Sea. Sci. China Earth Sci..

[68] Dai S, Zhao YF, Liu HJ, Hu ZY, Zheng S, Zhu ML (2020). The effects of a warm-core eddy on chlorophyll *a* distribution and phytoplankton community structure in the northern South China Sea in spring 2017. J. Mar. Syst..

[69] He XQ, Xu DF, Bai Y, Pan DL, Chen CA, Chen XY (2016). Eddy-entrained Pearl River plume into the oligotrophic basin of the South China Sea. Cont. Shelf Res..

[70] Pan XJ, Wong GTF, Ho TY, Tai JH, Liu HB, Liu JJ (2018). Remote sensing of surface [nitrite + nitrate] in river-influenced shelf-seas: The northern South China Sea Shelf-sea. Remote Sens. Environ..

[71] Xu J, Yin KD, He L, Yuan XC, Ho AYT, Harrison PPJ (2008). Phosphorus limitation in the northern South China Sea during late summer: Influence of the Pearl River. Deep-Sea Res. I..

[72] Caron D (1994). Inorganic nutrients, bacteria, and the microbial loop. Microb. Ecol..

[73] Kirchman D (1994). The uptake of inorganic nutrients by heterotrophic bacteria. Microb. Ecol..

[74] Song JM (2011). Biogeochemical Processes of Biogenic Elements in China Marginal Seas.

[75] Zhang WC, Chen X, Li HB, Zhao L, Zhao Y, Dong Y (2016). Review of nutrient (nitrogen and phosphorus) regeneration in the marine pelagic microbial food web. Mar. Sci. Bull..

[76] Ma J, Song JM, Li XG, Yuan HM, Li N, Duan LQ (2020). Effects of Y3 seamount on nutrients influencing the ecological environment in the Western Pacific Ocean. Earth Sci. Front..

[77] Li HB, Zhang WC, Zhao Y, Zhao L, Dong Y, Wang CF (2018). Tintinnid diversity in the tropical West Pacific Ocean. Acta Oceanol. Sin..

[78] Dolan JR, Ritchie ME, Ras J (2007). The, “neutral” community structure of planktonic herbivores, tintinnid ciliates of the microzooplankton, across the SE Tropical Pacific Ocean. Biogeosciences.

[79] Dolan JR, Ritchie ME, Tunin-Ley A, Pizay M (2009). Dynamics of core and occasional species in the marine plankton: Tintinnid ciliates in the north-west Mediterranean Sea. J. Biogeogr..

[80] Dolan JR, Marrasé C (1995). Planktonic ciliate distribution relative to a deep chlorophyll maximum: Catalan Sea, NW Mediterranean, June 1993. Deep-Sea Res..

[81] Suzuki T, Taniguchi A (1998). Standing crops and vertical distribution of four groups of marine planktonic ciliates in relation to phytoplankton chlorophyll *a*. Mar. Biol..

[82] Utermöhl H (1958). Zur vervollkommnung der quantitativen phytoplankton Methodik. Mit. Int. Ver. Theor. Angew. Limnol..

[83] Lund JWG, Kipling C, Cren EDL (1958). The inverted microscope method of estimating algal numbers and the statistical basis of estimations by counting. Hydrobiologia.

[84] Kofoid CA, Campbell AS (1929). A Conspectus of the Marine and Fresh-Water Ciliata Belonging to the Suborder Tintinnoinea: With Descriptions of New Species Principally from the Agassiz Expedition to the Eastern Tropical Pacific 1904–1905.

[85] Kofoid, C. A., & Campbell, A. S. Reports on the scientific results of the expedition to the eastern tropical Pacific, in charge to Alexander Agassiz, by US Fish commission steamer “Albatross”, from October 1904 to March 1905, The Ciliata: The Tintinnoinea (Bulletin of the Museum of Comparative Zoology of Harvard College), vol. XXXVII. Cambridge University, Harvard (Lieut.-Commander LM Garrett, USN commanding) (1939).

[86] Zhang WC, Feng MP, Yu Y, Zhang CX, Xiao T (2012). An Illustrated Guide to Contemporary Tintinnids in the World.

[87] Paranjape MA, Gold K (1982). Cultivation of marine pelagic protozoa. Ann. Inst. Oceanogr. Paris.

[88] Alder VA, Boltovskoy D (1999). Tintinnoinea. South Atlantic zooplankton.

[89] Verity PG, Langdon C (1984). Relationships between lorica volume, carbon, nitrogen, and ATP content of tintinnids in Narragansett Bay. J. Plankton R..

[90] Putt M, Stoecker DK (1989). An experimentally determined carbon: Volume ratio for marine “oligotrichous” ciliates from estuarine and coastal waters. Limnol. Oceanogr..

[91] Yu Y, Zhang WC, Zhang CX, Zhou F, Zhao N, Xiao T (2014). Basin-scale variation in planktonic ciliate distribution: A detailed temporal and spatial study of the Yellow Sea. Mar. Biol. Res..

[92] Wang CF, Yang MY, He Y, Xu ZQ, Zhao Y, Zhang WC (2022). Hydrographic feature variation caused pronounced differences of planktonic ciliate community in the Pacific Arctic Region in summer 2016 and 2019. Front. Microbiol..

[93] Margalef R (1958). Information theory in ecology. Gen. Syst..

[94] Shannon CE (1948). A mathematical theory of communication. Bell Syst. Tech. J..

[95] Dolan JR, Pierce RW, Dolan JR, Agatha S, Coats DW (2013). Diversity and distributions of tintinnid ciliates. The Biology and Ecology of Tintinnid Ciliates: Models for Marine Plankton.

[96] Xu ZL, Chen YQ (1989). Aggregated intensity of dominant species of zooplankton in autumn in the East China Sea. J. Ecol..

[97] Anderson MJ, Gorley RN, Clarke KR (2008). PERMANOVA+ for PRIMER: Guide to Software and Statistical Methods.

[98] Jiang Y, Xu G, Xu H (2016). Use of multivariate dispersion to assess water quality based on species composition data. Environ. Sci. Pollut. Res..

