# Supplementary material for: Diel variations in planktonic ciliate community structure in the northern South China Sea and tropical Western Pacific
Source: Sci Rep. 2023 Mar 8;13:3843. doi: 10.1038/s41598-023-30973-6 (PMC9995376; doi:10.1038/s41598-023-30973-6)
Supplement: Supplementary file 1 — Supplementary Information 1. [file 41598_2023_30973_MOESM1_ESM.pdf]

Supplemental material figures

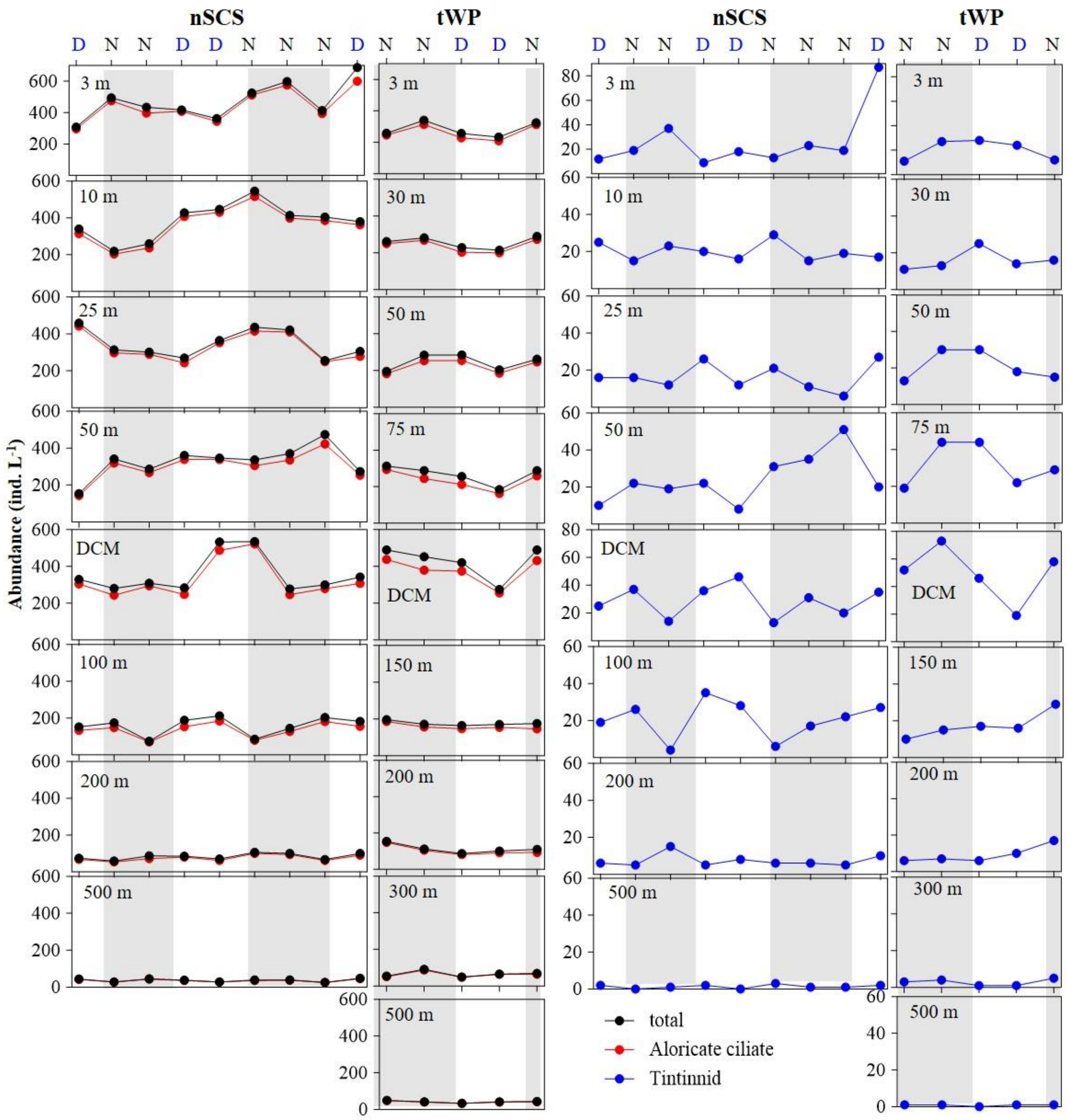

Supplementary Figure 1. Day (D)-night (N) variations of ciliate (total, aloricate ciliate and tintinnid) abundance at each depth in the nSCS and tWP. Black shadows: night.

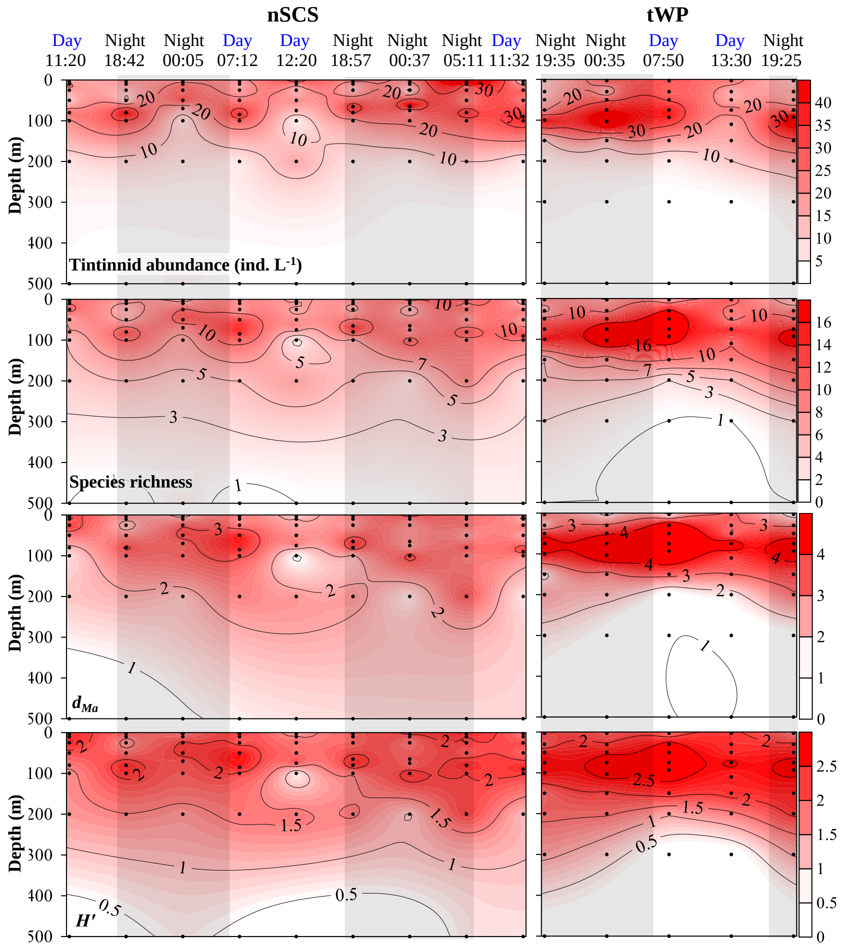

Supplementary Figure 2. Diel variations of tintinnid abundance, species richness and diversity indices at each layers in the nSCS and tWP. d_{Ma}: Margalef index; H': Shannon index. Black shadows: night.

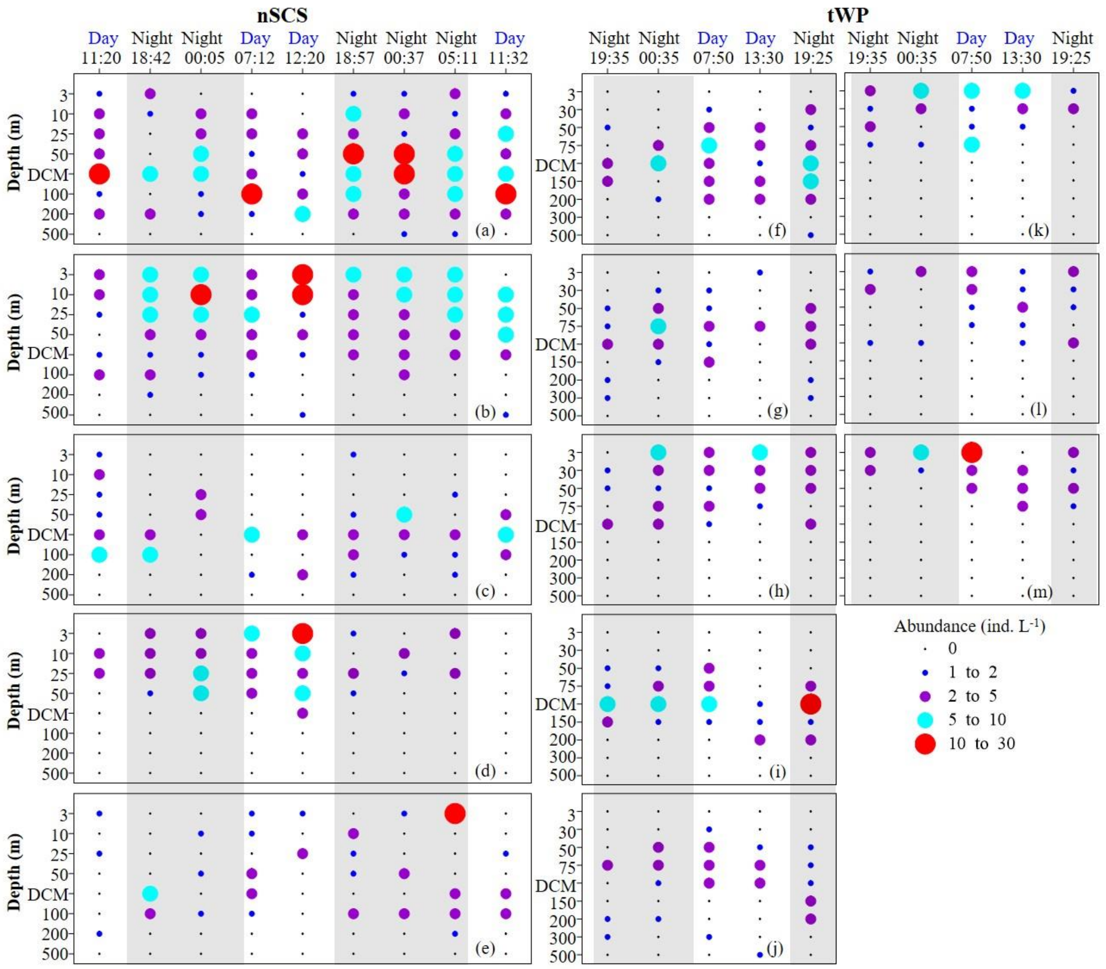

Supplementary Figure 3. Diel variations of tintinnid dominant species at each layers in the nSCS and tWP. (a) and (f): *Salpingella faurei*; (b): *Dadayiella ganymedes*; (c) and (g): *Proplectella perpusilla*; (d): *Steenstrupiella steenstrupii*; (e): *Epiplocylis acuminata*; (h): *Ascampbelliella armilla*; (i): *Acanthostomella minutissima*; (j): *Eutintinnus hasleae*; (k): *Canthariella brevis*; (l): *Metacylis sanyahensis*; (m): *Protorhabdonella curta*. Black shadows: night.

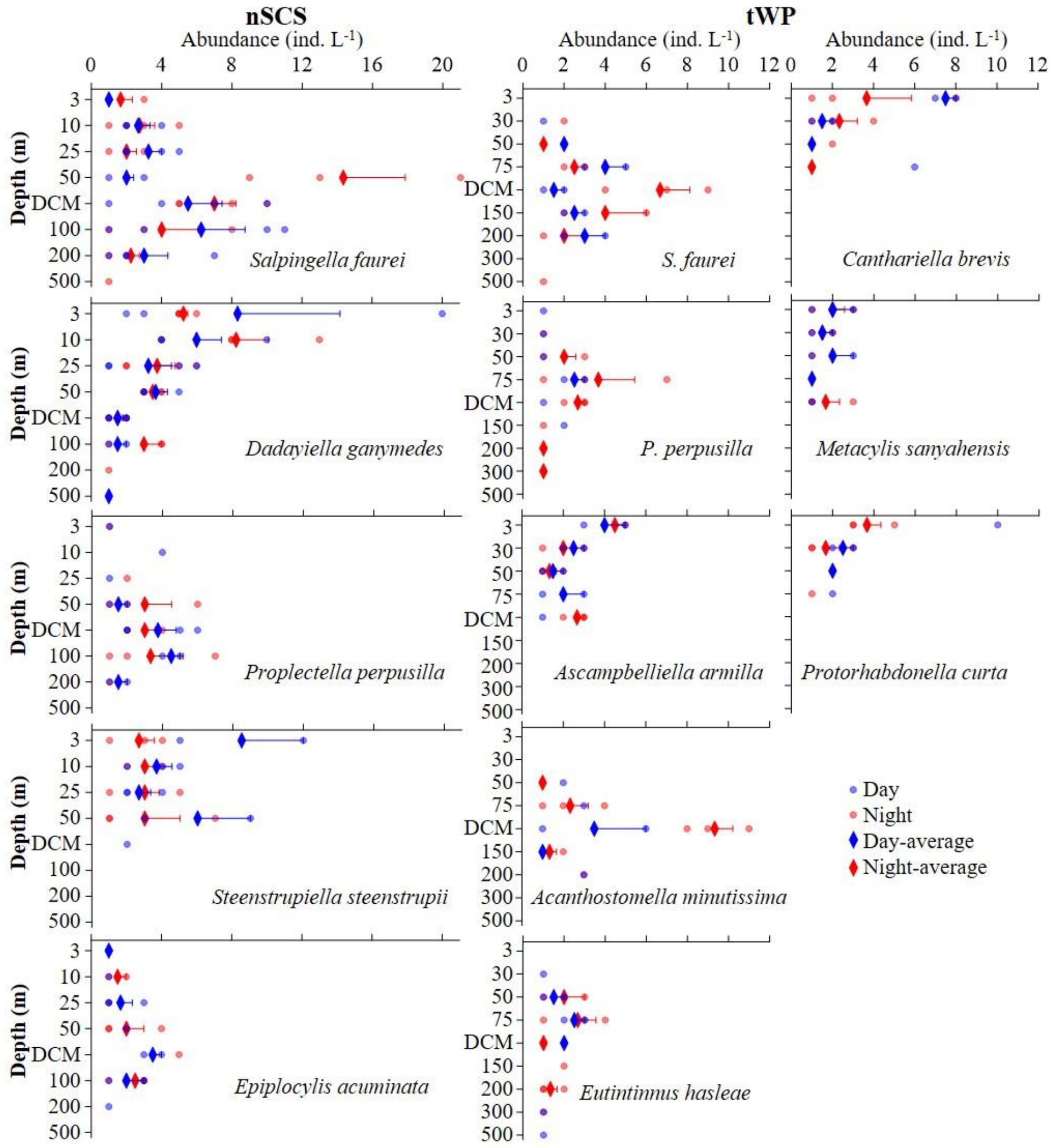

Supplementary Figure 4. Diel variations of vertical distribution of tintinnid dominant species (average) abundance from surface to 500 m in the nSCS and tWP.

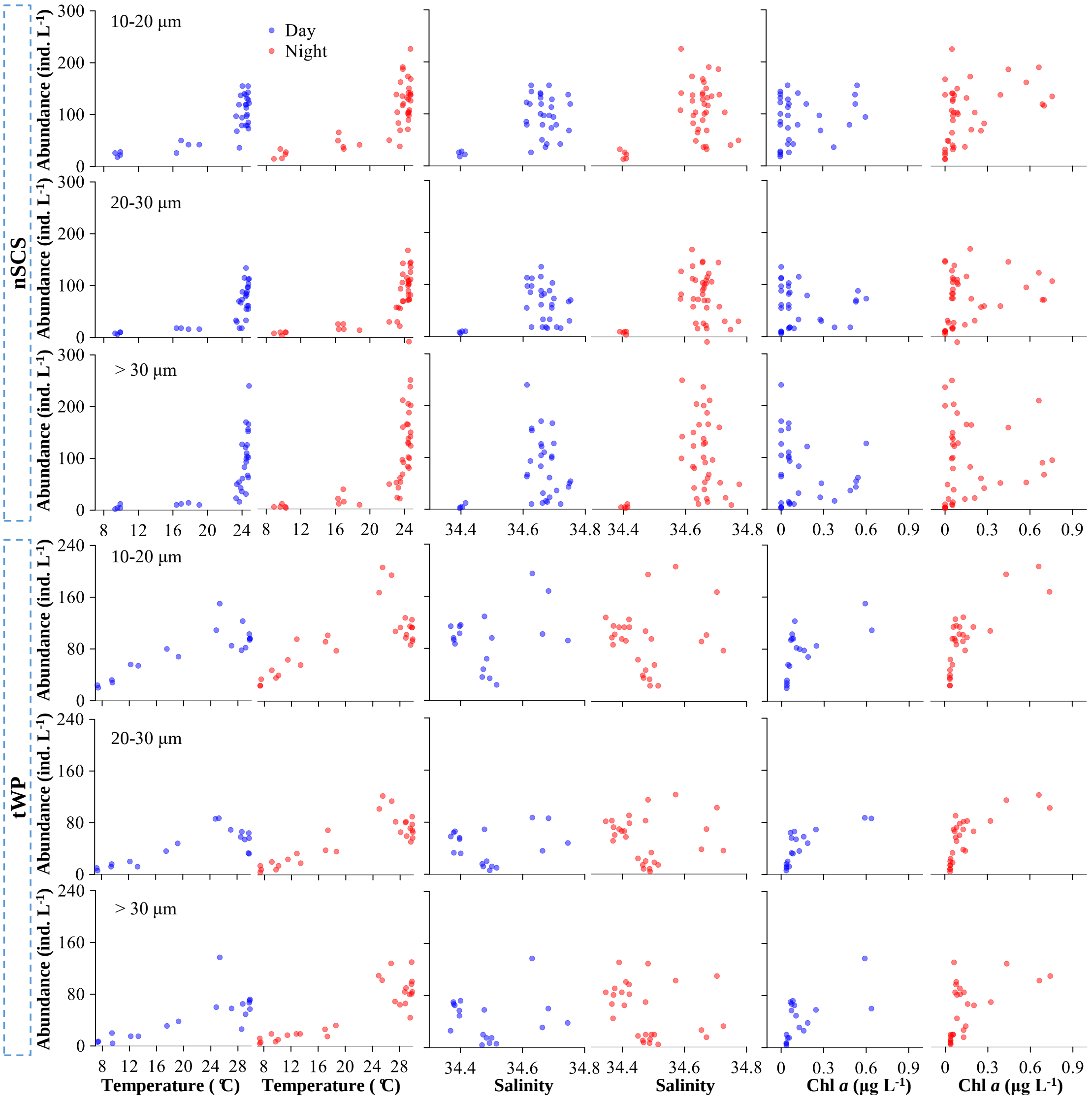

Supplementary Figure 5. Relationship between aloricate ciliate abundance and temperature, salinity, Chl *a* in the nSCS and tWP.

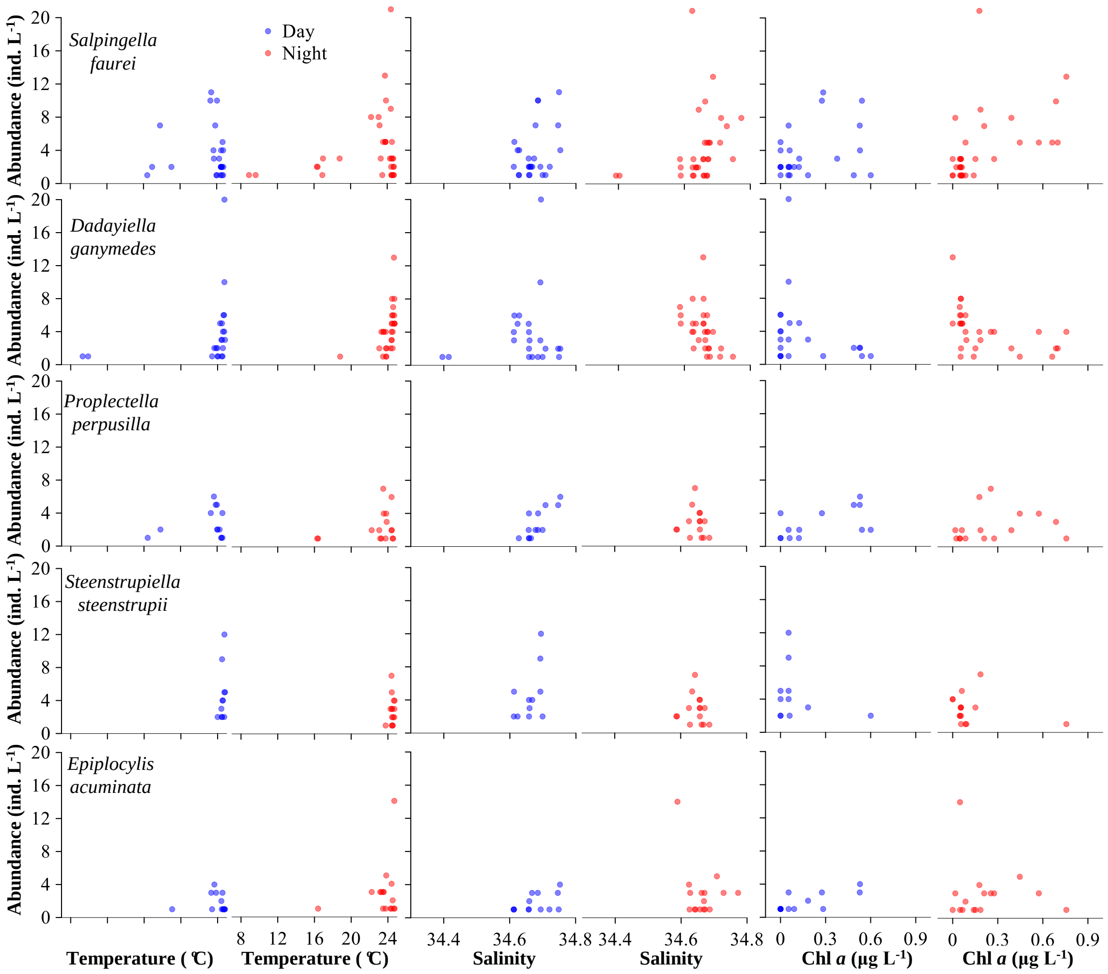

Supplementary Figure 6. Relationship between tintinnid dominant species abundance and temperature, salinity, Chl *a* in the nSCS.

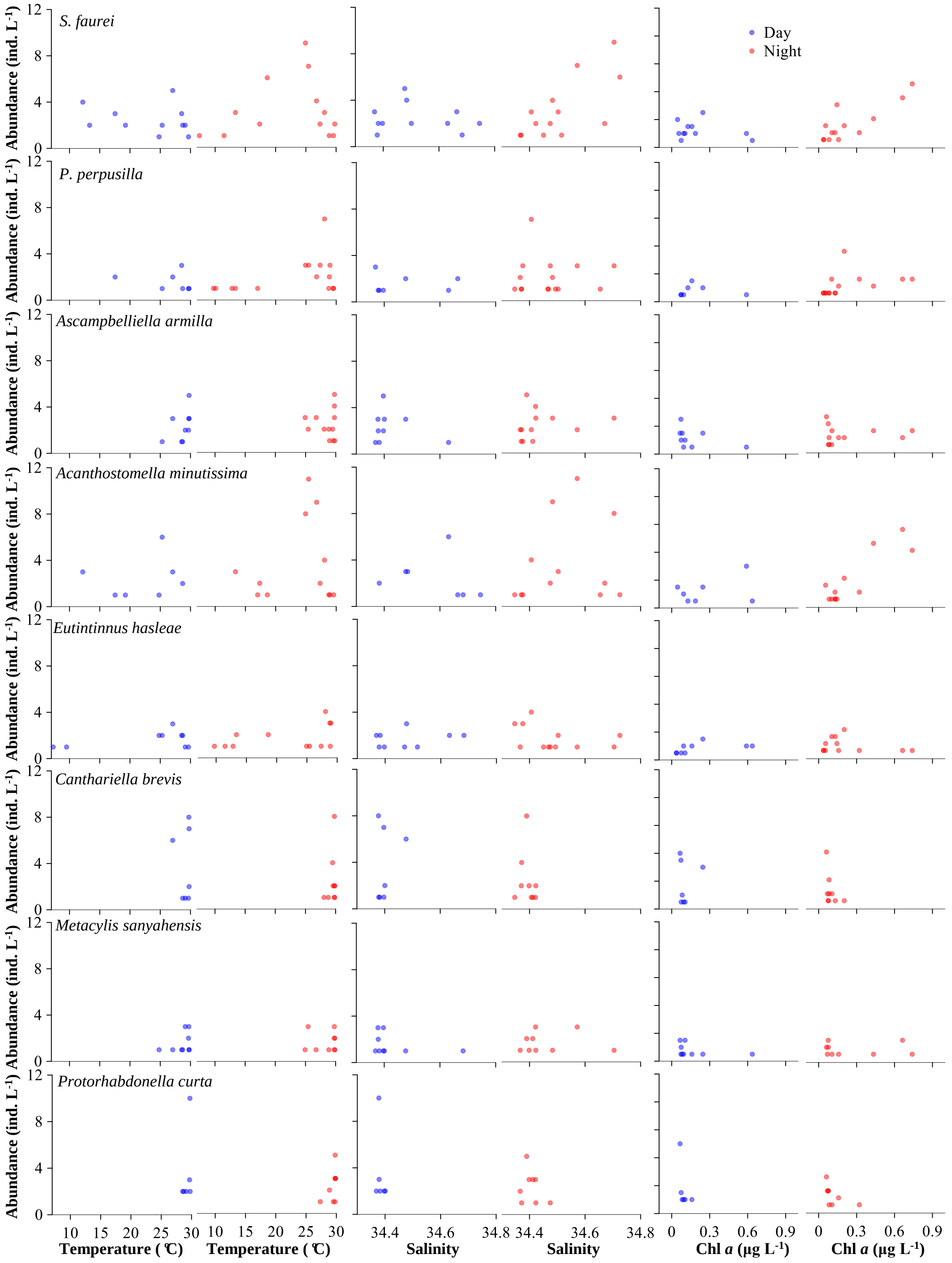

Supplementary Figure 7. Relationship between tintinnid dominant species abundance and temperature, salinity, Chl *a* in the tWP.

Supplemental material tables

Table S1 Results of PERMANOVA based on Bray Curtis similarity matrices derived from Square root-transformed abundance data of ciliate total abundance between day and night in the northern South China Sea (nSCS) and tropical Western Pacific (tWP).

**Seas**	**PERMANOVA between total ciliates**	**df**	**Sum of Sqs**	**R^2**	**Pseudo-*F***	***P***
nSCS	Groups	1	0.009	0.001	0.074	0.944
	Residual	70	8.348	0.999		
	Total	71	8.357	1.000		
tWP	Groups	1	0.059	0.014	0.609	0.494
	Residual	43	4.145	0.986		
	Total	44	4.204	1.000		

Table S2 Tintinnid species dimensions (LL: lorica length, μm; LOD: lorica oral diameter, μm), biogeography type (BT), maximum abundance (A_{max}, ind. L^{-1}), occurrence frequency (OF, %) and its dominance index (*Y*) at day and night in the northern South China Sea (nSCS) and tropical West Pacific (tWP).

**Species**	**LL**	**LOD**	**BT**	**nSCS-Day**			**nSCS-Night**			**tWP-Day**			**tWP-Night**		
				A_{max}	OF	*Y*	A_{max}	OF	*Y*	A_{max}	OF	*Y*	A_{max}	OF	*Y*
Acanthostomella conicoides	38.41 ±2.02	20.14 ±1.21	C	1	12.5	<0.01	4	15.00	<0.01	3	22.22	<0.01	8	22.22	<0.01
*A. lata**	36.86 ±1.60	28.09 ±1.18	C	-	-	-	-	-	-	3	5.56	<0.01	5	7.41	<0.01
A. minutissim	25.58 ±3.14	18.84 ±1.11	C	-	-	-	-	-	-	**6**	**38.89**	**0.02**	**11**	**44.44**	**0.04**
Amphorellopsis acantharus	60.82 ±3.14	24.92 ±0.51	C	-	-	-	-	-	-	4	16.67	<0.01	7	14.81	<0.01
*Amphorides amphora**	92.80 ±10.37	39.89 ±2.70	C	3	28.13	<0.01	4	20.00	<0.01	1	11.11	<0.01	1	22.22	<0.01
*A. laackmanni**	86.93 ±8.72	25.90 ±0.96	C	-	-	-	2	5.00	<0.01	-	-	-	-	-	-
A. minor	69.78 ±2.01	36.10 ±1.19	C	1	3.13	<0.01	1	2.50	<0.01	1	11.11	<0.01	3	22.22	<0.01
A. quadrilineata	113.41 ±13.57	48.26 ±3.50	C	3	12.5	<0.01	4	15.00	<0.01	1	5.56	<0.01	2	7.41	<0.01
Ascampbelliella armilla	29.73 ±1.81	22.37 ±1.18	W	1	12.5	<0.01	4	12.50	<0.01	**5**	**50.00**	**0.04**	**5**	**44.44**	**0.03**
*A. retusa**	33.65 ±3.46	30.00 ±0.96	W	-	-	-	4	5.00	<0.01	2	16.67	<0.01	4	18.52	<0.01
*Brandtiella palliata**	146.75 ±4.35	46.26 ±2.46	W	-	-	-	-	-	-	-	-	-	2	11.11	<0.01
Canthariella brevis	64.94 ±3.11	29.69 ±1.66	W	2	9.38	<0.01	3	7.50	<0.01	**8**	**38.89**	**0.03**	8	33.33	<0.02
*C. pyramidata**	52.71 ±2.20	27.46 ±0.79	W	-	-	-	1	2.50	<0.01	3	5.56	<0.01	1	14.81	<0.01
*C. truncata**	49.08 ±2.47	27.57 ±1.06	W	-	-	-	-	-	-	-	-	-	1	3.70	<0.01
*Climacocylis scalaria**	222.41 ±45.54	54.01 ±5.88	W	3	3.13	<0.01	1	7.50	<0.01	1	11.11	<0.01	-	-	-
*Codonella grahami**	65.80 ±0.74	47.48 ±0.31	C	-	-	-	2	2.50	<0.01	-	-	-	-	-	-
*Codonellopsis meridionalis**	182.71 ±10.47	58.91 ±1.19	C	-	-	-	-	-	-	1	5.56	<0.01	1	3.70	<0.01
*C. morchella**	89.62 ±11.28	33.75 ±0.94	C	-	-	-	2	10.00	<0.01	-	-	-	-	-	-
*C. robusta**	67.40 ±10.24	33.12 ±1.66	C	-	-	-	1	2.50	<0.01	1	5.56	<0.01	-	-	-
*Coxliella laciniosa**	83.16 ±8.08	60.09 ±0.57	-	1	3.13	<0.01	-	-	-	1	11.11	<0.01	1	3.70	<0.01
*Cyttarocylis acutiformis**	228.29	124.69	W	-	-	-	-	-	-	1	5.56	<0.01	-	-	-

Dadayiella ganymedes	92.40 ±12.87	28.95 ±1.85	C	**20**	**68.75**	**0.11**	**13**	**72.50**	**0.12**	2	27.78	<0.01	4	25.93	<0.01
D. pachytoecus[*]	96.72 ±4.04	32.23 ±1.52	C	1	6.25	<0.01	1	5.00	<0.01	-	-	-	-	-	-
Daturella striata[*]	244.36	59.58	W	1	3.13	<0.01	-	-	-	-	-	-	-	-	-
Dictyocysta reticulata	58.29 ±2.55	38.42 ±0.82	C	2	12.5	<0.01	9	15.00	<0.01	3	5.56	<0.01	1	3.70	<0.01
D. spinosa[*]	52.53 ±1.54	35.49 ±1.60	C	1	6.25	<0.01	1	7.50	<0.01	-	-	-	-	-	-
Epiplocylis acuminata	70.16 ±4.93	50.27 ±2.68	W	4	40.63	<0.02	**14**	**40.00**	**0.02**	1	16.67	<0.01	2	11.11	<0.01
E. constricta	109.09 ±7.59	59.65 ±1.40	W	2	12.5	<0.01	6	5.00	<0.01	-	-	-	1	11.11	<0.01
E. undella[*]	155.44 ±14.14	72.05 ±1.22	W	-	-	-	-	-	-	2	5.56	<0.01	-	-	-
Eutintinnus apertus	58.01 ±13.45	26.89 ±1.63	C	3	25	<0.01	4	30.00	<0.01	2	33.33	0.01	5	29.63	<0.01
E. fraknoii	248.43 ±27.90	46.46 ±1.94	C	1	15.63	<0.01	1	7.50	<0.01	2	22.22	<0.01	1	11.11	<0.01
E. hasleae	42.07 ±10.47	30.02 ±2.78	C	1	6.25	<0.01	-	-	-	**3**	**50.00**	**0.03**	**4**	**44.44**	**0.02**
E. lusus-undae	179.97 ±20.25	42.74 ±3.58	C	2	25	<0.01	6	30.00	<0.01	2	11.11	<0.01	2	18.52	<0.01
E. pacificus[*]	67.92 ±13.68	26.15 ±1.50	C	1	6.25	<0.01	-	-	-	2	11.11	<0.01	5	11.11	<0.01
E. stramentus[*]	165.01 ±20.05	24.66 ±1.42	C	-	-	-	1	2.50	<0.01	-	-	-	1	3.70	<0.01
E. tubulosus[*]	121.980	30.54	C	-	-	-	-	-	-	-	-	-	1	3.70	<0.01
Metacylis sanyahensis	24.90 ±4.53	23.53 ±1.48	C	-	-	-	1	2.50	<0.01	**3**	**50.00**	**0.02**	3	33.33	0.01
Ormosella bresslaui[*]	55.31	31.27	-	-	-	-	-	-	-	1	5.56	<0.01	-	-	-
Parundella aculeata	160.54 ±18.38	30.37 ±0.95	C	8	15.63	<0.01	4	25.00	<0.01	-	-	-	-	-	-
P. difficilis[*]	107.59 ±13.97	41.94 ±2.18	C	2	6.25	<0.01	1	2.50	<0.01	-	-	-	-	-	-
P. inflata	105.18 ±4.18	29.25 ±1.03	C	-	-	-	-	-	-	3	11.11	<0.01	7	18.52	<0.01
P. praetenuis[*]	124.25 ±2.93	32.68 ±0.17	C	4	3.13	<0.01	1	5.00	<0.01	-	-	-	-	-	-
Poroecus curtus[*]	62.46 ±1.07	28.11 ±0.91	W	1	3.13	<0.01	-	-	-	1	11.11	<0.01	-	-	-
Proplectella claparedei	67.11 ±2.87	43.52 ±2.01	W	-	-	-	1	2.50	<0.01	1	16.67	<0.01	1	14.81	<0.01
P. parva	56.38 ±2.93	27.77 ±0.61	W	7	9.38	<0.01	8	20.00	<0.01	1	5.56	<0.01	2	25.93	<0.01
P. perpusilla	44.62 ±4.67	28.78 ±0.72	W	**6**	**40.63**	**0.03**	**7**	**40.00**	**0.02**	3	38.89	0.01	**7**	**55.56**	**0.04**
P. urna[*]	33.12 ±2.59	14.90 ±0.68	W	1	6.25	<0.01	-	-	-	-	-	-	1	11.11	<0.01

Protorhabdonella curta	42.15 ±5.14	28.70 ±1.78	C	1	3.13	<0.01	1	5.00	<0.01	**10**	**33.33**	**0.02**	5	29.63	0.01
P. simplex	54.15 ±3.19	33.68 ±1.00	C	2	9.38	<0.01	10	12.50	<0.01	1	27.78	<0.01	3	22.22	<0.01
Rhabdonella amor	87.19 ±2.94	46.40 ±1.77	W	1	9.38	<0.01	12	5.00	<0.01	-	-	-	1	7.41	<0.01
R. conica[*]	386.31	60.37	W	-	-	-	1	5.00	<0.01	-	-	-	-	-	-
R. cornucopia[*]	134.39 ±8.23	51.23 ±2.85	W	-	-	-	8	12.50	<0.01	-	-	-	-	-	-
R. elegans[*]	169.05	44.96	W	1	3.13	<0.01	-	-	-	-	-	-	-	-	-
R. exilis	67.07 ±5.14	28.63 ±1.51	W	2	3.13	<0.01	3	12.50	<0.01	1	22.22	<0.01	1	22.22	<0.01
R. poculum[*]	86.50 ±1.31	47.4 ±0.51	W	1	6.25	<0.01	12	5.00	<0.01	-	-	-	-	-	-
R. sanyahensis	131.57 ±7.66	37.63 ±6.19	W	2	12.5	<0.01	1	2.50	<0.01	-	-	-	-	-	-
Rhabdonellopsis apophysata	279.46 ±15.06	57.38 ±2.35	W	1	6.25	<0.01	-	-	-	2	5.56	<0.01	1	11.11	<0.01
Salpingella acuminata	250.35 ±50.31	30.35 ±3.28	C	5	15.63	<0.01	4	17.50	<0.01	2	27.78	<0.01	4	29.63	<0.01
S. curta	95.56 ±9.90	14.01 ±0.64	C	3	15.63	<0.01	5	20.00	<0.01	4	27.78	<0.01	3	29.63	0.01
S. decurtata[*]	147.27 ±16.45	17.33 ±1.85	C	4	18.75	<0.01	5	12.50	<0.01	1	27.78	<0.01	2	22.22	<0.01
S. faurei	121.11 ±15.72	13.05 ±0.87	C	**11**	**78.13**	**0.13**	**21**	**82.50**	**0.15**	**5**	**61.11**	**0.06**	**9**	**48.15**	**0.04**
S. minutissima[*]	77.51 ±7.73	13.33 ±0.52	C	-	-	-	2	5.00	<0.01	-	-	-	-	-	-
S. rotundata[*]	98.08 ±14.36	14.21 ±1.97	C	4	3.13	<0.01	1	2.50	<0.01	-	-	-	-	-	-
Steenstrupiella gracilis	76.77 ±3.67	31.48 ±1.93	C	4	34.38	<0.02	6	40.00	<0.02	3	33.33	<0.01	2	25.93	<0.01
S. intumescens	206.20 ±16.10	36.52 ±1.62	C	2	12.5	<0.01	2	7.50	<0.01	4	27.78	<0.01	3	14.81	<0.01
S. robusta	113.78 ±12.94	34.60 ±1.85	C	8	31.25	<0.02	6	12.50	<0.01	-	-	-	-	-	-
S. steenstrupii	153.72 ±12.03	35.07 ±1.58	C	**12**	**34.38**	**0.03**	**7**	**37.50**	**0.02**	2	22.22	<0.01	3	3.70	<0.01
Xystonellopsis brandti[*]	201.57 ±7.81	33.35 ±1.07	W	-	-	-	-	-	-	1	5.56	<0.01	1	3.70	<0.01
X. favata	233.88	61.79	W	-	-	-	1	2.50	<0.01	-	-	-	-	-	-

Note: The biogeography of tintinnid genera (e.g. cosmopolitan and warm water types) were derived according to Dolan et al. (2013); [*]: species with counting number <10; Species in bold black were regarded as dominant species in an assemblage with $Y \geq 0.02$; The LL and LOD were presented as mean values ±standard deviation. C: Composition; W: Warm Water; -: not classified in Dolan et al. (2013).

Table S3 Day-night variations in tintinnid species genera, species richness, biogeography type (BT) and its percentage (BTP, %) in the northern South China Sea (nSCS) and tropical West Pacific (tWP).

**Seas**	**Day/Night**	**Genera**	**Species richness**	**BT**			**BTP**		
				C	W	-	C	W	-
nSCS	Day	19	44	27	16	1	61.36	36.36	2.28
	Night	19	49	31	18	0	63.27	36.73	0
	All	23	57	34	22	1	59.65	38.6	1.75
tWP	Day	24	44	27	15	2	61.36	34.09	4.55
	Night	21	45	28	16	1	62.22	35.56	2.22
	All	25	51	29	20	2	56.86	39.22	3.92
Total		27	69	40	27	2	57.97	39.13	2.9

Table S4 Spearman's rank correlation between the planktonic ciliate (aloricate size-fraction and tintinnid dominant species) abundance (ind. L^{-1}) and depth (m), temperature (T, ℃), salinity, and chlorophyll *a* concentrations (Chl *a*, μg L^{-1}).

Seas	Group	Size-fraction/Species	Day				Night			
			Depth	T	Salinity	Chl *a*	Depth	T	Salinity	Chl *a*
nSCS	Aloricate ciliate	10-20 μm	-0.728**	0.655**	0.558**	0.312	-0.748**	0.763**	0.570**	0.305
		20-30 μm	-0.820**	0.761**	0.574**	0.159	-0.827**	0.836**	0.573**	0.134
		> 30 μm	-0.899**	0.830**	0.548**	0.051	-0.874**	0.858**	0.563**	0.075
		All	-0.847**	0.823**	0.573**	0.194	-0.842**	0.805**	0.573**	0.154
	Tintinnid	*Salpingella faurei*	0.017	0.049	0.520**	0.297	-0.066	0.143	0.441**	0.448**
		Dadayiella ganymedes	-0.651**	0.655**	0.283	0.010	-0.867**	0.831**	0.482**	-0.013
		Proplectella perpusilla	0.080	-0.034	0.300	0.548**	0.130	-0.047	0.297	0.178
		Steenstrupiella steenstrupii	-0.585**	0.515**	0.266	-0.115	-0.673**	0.563**	0.283	-0.173
		Epiplocylis acuminata	-0.234	0.267	0.302	0.186	-0.052	0.124	0.297	-0.015
		All	-0.384*	0.453**	0.574**	0.471**	-0.558**	0.596**	0.574**	0.344*
	Total		-0.838**	0.819**	0.573**	0.215	-0.844**	0.805**	0.573**	0.154
tWP	Aloricate ciliate	10-20 μm	-0.819**	0.836**	0.087	0.538*	-0.703**	0.715**	0.115	0.569**
		20-30 μm	-0.709**	0.768**	0.110	0.606**	-0.756**	0.816**	0.040	0.485*
		> 30 μm	-0.675**	0.722**	0.078	0.591**	-0.762**	0.864**	-0.179	0.347
		All	-0.776**	0.817**	0.095	0.604**	-0.770**	0.828**	-0.005	0.491**
	Tintinnid	*S. faurei*	-0.491*	0.522*	-0.398	-0.199	-0.429*	0.467*	-0.283**	-0.136**
		P. perpusilla	-0.387	0.388	-0.078	0.020	-0.107	0.114	0.040	0.406*
		Ascampbelliella armilla	-0.653**	0.732**	-0.483*	-0.161	-0.547**	0.629**	-0.148	0.280
		Acanthostomella minutissima	-0.181	0.116	0.249	0.572*	-0.195	0.220	0.420*	0.759**
		Eutintinnus hasleae	-0.228	0.376	0.000	0.431	-0.199	0.181	0.043	0.057
		Canthariella brevis	-0.841**	0.800**	-0.544*	-0.272	-0.768**	0.776**	-0.409*	-0.195
		Metacylis sanyahensis	-0.364**	0.420**	-0.088	-0.044	-0.279*	0.333**	-0.002	0.337*
		Protorhabdonella curta	-0.434	0.485*	-0.349	-0.175	-0.497**	0.528**	-0.320	-0.153
		All	-0.725**	0.766**	-0.021	0.386	-0.452*	0.490**	0.316	0.720**
	Total		-0.778**	0.820**	0.080	0.583*	-0.747**	0.804**	0.040	0.537**

Note: **: $p<0.01$, *: $p<0.05$

Table S5 Sampling stations location, sampling time and day/night classification in the northern South China Sea (nSCS) and tropical West Pacific (tWP).

Seas	stations	Latitude (°N)	Longitude (°E)	Date	Time	Day/Night
nSCS	TS1	19.8531	116.1238	2017.03.29	11:20	Day
	TS2	19.8531	116.1238	2017.03.29	18:42	Night
	TS3	19.8531	116.1238	2017.03.30	0:05	Night
	TS4	19.8531	116.1238	2017.03.30	7:12	Day
	TS5	19.8531	116.1238	2017.03.30	12:20	Day
	TS6	19.8531	116.1238	2017.03.30	18:57	Night
	TS7	19.8531	116.1238	2017.03.31	0:37	Night
	TS8	19.8531	116.1238	2017.03.31	5:11	Night
	TS9	19.8531	116.1238	2017.03.31	11:32	Day
tWP	TS1	10.0778	140.1889	2019.06.02	19:35	Night
	TS2	10.0778	140.1889	2019.06.03	0:35	Night
	TS3	10.0778	140.1889	2019.06.03	7:50	Day
	TS4	10.0778	140.1889	2019.06.03	13:30	Day
	TS5	10.0778	140.1889	2019.06.03	19:25	Night
